# Parcellation of the cingulate cortex at rest and during tasks: a meta-analytic clustering and experimental study

**DOI:** 10.3389/fnhum.2013.00275

**Published:** 2013-06-14

**Authors:** Diana M. E. Torta, Tommaso Costa, Sergio Duca, Peter T. Fox, Franco Cauda

**Affiliations:** ^1^Department of Psychology, Università di TorinoTorino, Italy; ^2^CCS fMRI-Brain Connectivity and Complex Systems Unit, Koelliker HospitalTorino, Italy; ^3^Research Imaging Institute, University of Texas Health Science Center at San AntonioSan Antonio, TX, USA

**Keywords:** meta-analytic clustering, voxel-based meta-analysis, meta-analytic connectivity modeling, activation likelihood estimation, data driven parcellation, k-means clustering, hierarchical clustering, voronoi parcellation

## Abstract

Anatomical, morphological, and histological data have consistently shown that the cingulate cortex can be divided into four main regions. However, less is known about parcellations of the cingulate cortex when involved in active tasks. Here, we aimed at comparing how the pattern of clusterization of the cingulate cortex changes across different levels of task complexity. We parcellated the cingulate cortex using the results of a meta-analytic study and of three experimental studies. The experimental studies, which included two active tasks and a resting state protocol, were used to control the results obtained with the meta-analytic parcellation. We explored the meta-analytic parcellation by applying a meta-analytic clustering (MaC) to papers retrieved from the BrainMap database. The MaC is a meta-analytic connectivity driven parcellation technique recently developed by our group which allowed us to parcellate the cingulate cortex on the basis of its pattern of co-activations during active tasks. The MaC results indicated that the cingulate cortex can be parcellated into three clusters. These clusters covered different percentages of the cingulate parenchyma and had a different density of foci, with the first cluster being more densely connected. The control experiments showed different clusterization results, suggesting that the co-activations of the cingulate cortex are highly dependent on the task that is tested. Our results highlight the importance of the cingulate cortex as a hub, which modifies its pattern of co-activations depending on the task requests and on the level of task complexity. The neurobiological meaning of these results is discussed.

## Introduction

The cingulate cortex is the thick part of the cerebral cortex surrounding the corpus callosum and is currently thought to be made up of four subregions (the four-region neurobiological model): The anterior cingulate cortex, which includes the perigenual cingulate cortex, the midcingulate cortex, the posterior cingulate cortex and the restrosplenial cingulate cortex (Vogt, 2009). Such four major subdivisions were built mainly on structural observations (Vogt and Pandya, 1987; Vogt et al., 1987, 1992, 2001, 2003; Vogt and Derbyshire, 2002; Vogt and Vogt, 2003; Vogt and Laureys, 2005; Fan et al., 2008; Palomero-Gallagher et al., 2008, 2009; Vogt, 2009) and were proposed to subserve specific functions, with the anterior cingulate cortex involved in affective evaluation (Allman et al., 2001), conflict monitoring (Carter et al., 1998, 1999; Botvinick et al., 2004), error monitoring and detection (Holroyd et al., 1998; Gehring and Knight, 2000; Gehring and Fencsik, 2001), response selection (Paus et al., 1993; Awh and Gehring, 1999) and attention control (Posner and Dehaene, 1994; Crottaz-Herbette and Menon, 2006); the midcingulate cortex involved in cognitive tasks such as attention for action (Pardo et al., 1990; Badgaiyan and Posner, 1998), response selection (Corbetta et al., 1991; Paus et al., 1993), error detection and competition monitoring (Carter et al., 1998), anticipation (Murtha et al., 1996), and working memory (Petit et al., 1998); the posterior cingulate cortex in tasks related to visuospatial orientation and navigation of the body in environmental space (Vogt and Laureys, 2005), self-reflection and autobiographical memory (Spreng et al., 2009); and finally, the retrosplenial cingulate cortex in memory and visuospatial functions (Vogt and Pandya, 1987; Vogt et al., 1987; Parker and Gaffan, 1997; Burgess et al., 2001; Vogt, 2005; Iaria et al., 2007; Keene and Bucci, 2008; Vann et al., 2009). Until recently this “segregationist model,” for which each portion of the cingulate cortex subserves specific functions, has been adopted.

Recent studies have used magnetic resonance imaging (MRI) or functional MRI (fMRI) to investigate the connectivity based parcellation of the cingulate cortex. Beckmann and colleagues (2009) used probabilistic diffusion tractography (a measure of anatomical connectivity), to characterize the cingulate probabilistic connectivity. By applying a connectivity-based parcellation, they found nine distinct clusters in the cingulate cortex (Beckmann et al., 2009). Yu and colleagues investigated the functional connectivity of each of the subregions proposed in the four-region model and showed that each subregion was characterized by a specific pattern of functional connectivity (Yu et al., 2011). This finding led the authors to conclude that the anatomical segregation is confirmed by functional segregation (Margulies et al., 2007; Yu et al., 2011). While the functional connectivity of some specific subregions of the cingulate cortex during rest has been progressively disclosed (Koski and Paus, 2000; Margulies et al., 2007; Yu et al., 2011), to date far less is known about the functional profile and the parcellation of the cingulate cortex during tasks.

The advent of databases such as BrainMap (Fox et al., 2005; Laird et al., 2005a,b, 2009a,b), which stores thousands of fMRI experiments together with their results, has allowed performing the so-called voxel-based meta-analyses. Using these databases, many different studies can be pooled together to investigate the functional properties of specific brain regions when involved in active tasks. As a result of this approach, recent views have proposed that the same subregions of the cingulate cortex are involved in different functions (“integrationist model”). For example, Shackman and colleagues (2011) observed that in the rostral anterior cingulate cortex, pain, affect, and cognition overlap. Similarly, Torta and Cauda (2011) have reported that the midcingulate cortex is recruited in a variety of tasks ranging from pain to affect to attention and motor tasks.

Altogether these findings suggest that fMRI parcellations of the cingulate cortex lead to different results depending on the methodological approach. In addition, it has been shown that high-dimensional parcellations support the view of a hierarchical nested structure within the hub regions of the brain (Leech et al., 2011). Here, we aimed at comparing how the pattern of clusterization of the cingulate cortex changes across different levels of task complexity (i.e., tasks and non-tasks such as resting state). We performed a meta-analytic study using a meta-analytic tool recently developed by our group: the meta-analytic clustering (MaC) (Cauda et al., 2012). This method permits a voxel-wise data-driven clusterization of the patterns of co-activation of the cingulate cortex during the widest number of active tasks (Cauda et al., 2012). This technique starts from the meta-analytic data and produces a meta-analytic connectivity-based parcellation in a data-driven fashion (Torta and Cauda, 2011). We verified the results of the MaC study by performing three additional fMRI experiments on healthy volunteers. In this way we could observe possible modifications of the parcellation of the cingulate cortex during the widest number of active tasks, during specific tasks and during resting state.

For the control fMRI experiments, we scanned participants during resting state (experiment 2), during the presentation of emotional faces (experiment 3) and during the administration of painful stimuli (experiment 4). These latter two tasks were chosen as known to activate the cingulate cortex (Apkarian et al., 2005; Friebel et al., 2011). Subsequently we parcellated the cingulate cortex on the basis of the results of the two experiments and compared such findings to those obtained with the meta-analytic parcellation.

## Materials and methods

### Meta-analytic study

#### Database search

We queried the BrainMap database (Fox et al., 2005; Laird et al., 2005a,b, 2009a,b) for studies on healthy volunteers that recorded activations in the cingulate cortex. We did not select studies on the basis of the task performed as we were interested in investigating activations of the cingulate cortex in a broad range of cognitive tasks. Such a choice allowed us to characterize the parcellation of the cingulate cortex in a “working mode” irrespective of the task. The results of this search were saved in a series of files containing locations, papers and behavioral domains. For the meta-analysis, boundaries of the cingulate cortex were already coded in BrainMap, indeed we queried the database for all the papers that showed at least one focus in the cingulate cortex (http://www.brainmap.org/sleuth/). For subsequent analyses on in-house acquired fMRI data, the volume of interest (VOI) around the cingulate cortex was drawn by one of the authors (Franco Cauda), expert in neuroanatomical clustering, on the Colin 27 template (http://neuro.debian.net/pkgs/mni-colin27 nifti.html) at the group level. The MNI cingulate coordinates were then converted to Talairach coordinates using the icbm2Tal transform (http://brainmap.org/icbm2tal/).

### Meta-analytic clustering (MaC)

#### Data preparation

Here, we employed a methodology called MaC, delineated in a recent paper (Cauda et al., 2012). MaC works on meta-analytic connectivity modeling data (MACM; Robinson et al., 2010). Thus, as a first step, we performed a MACM. The MACM method is based on co-occurrences that are evaluated using the ALE approach. With a too small number of co-occurrences (i.e., foci) the ALE results are too dependent on the contribution of single foci (i.e., single studies) and are spatially very variable. As a consequence of this variability the parcellation results are unstable. Indeed the ALE method needs a minimum number of foci to produce a valid estimate (Laird et al., 2005a, 2009a; Eickhoff et al., 2009). To accomplish this need we created, in a completely unsupervised data-driven way, “blocks” of voxels each containing 50 foci.

The choice of 50 foci was supported by a simulation to evaluate the stability of the parcellation results using 10–100 foci in steps of 10. We parcellated the cingulate surface using blocks of different dimensions (namely with a different number of foci). For each dimension we repeated the entire process 100 times. We then checked the reliability of the results for each step evaluating if the results were stable for a given number of foci. The results were stable with blocks of n > 40 foci (see also Cauda et al., 2012) so we decided to opt for a minimum number of 50 foci as to further improve the stability obtained with 40 foci. We created blocks of voxels by employing the quad tree algorithm (Ballard and Brown, 1982). The quad tree is an algorithm that subdivides the two-dimensional space by decomposing the region into four equal quadrants and subsequently into four further subquadrants until each of the subquadrants contains the pre-defined number of foci (50 in our case). Thus, when the blocks meet the criterion, they are not subdivided further. In contrast, if more than 50 foci are found in a block, the algorithm further subdivides the block until the criterion of homogeneity is reached. As a result, we obtained 37 blocks containing 50 foci each but having different sizes. The quad tree decomposition returns a structure consisting of the *y* and *z* Talairach coordinates of each block and the corresponding papers in which Talairach coordinates of the foci appear. All parcellations were performed on a 3D mesh. To build the 3D mesh, a constant gray matter (GM) thickness was assumed, to allow subsequent methods to operate in 2D. Indeed, the 3D data from the database were projected to a plane passing through the coordinates *X* = 0 and then analyzed. For visualization purposes these data were back projected to a renderized brain surface on the basis of each voxel's original coordinate. All foci were projected within +1 and −5 mm from the GM plane to this surface. All these clustering analyses were performed using custom developed MATLAB scripts (Mathworks, Natick, MA, USA).

We calculated the MACM using the ALE algorithm (Laird et al., 2005a; Eickhoff et al., 2009) to pool the active foci of each quad tree. Each coordinate (focus) was modeled by a 3-D Gaussian distribution, defined by a full-width half-maximum (FWHM) of 10 mm (Turkeltaub et al., 2002). The ALE statistic was computed at every voxel in the brain. We made a valid assessment of the significance of the result by testing the values from the ALE images against null distributions. A threshold was applied, while controlling the false discovery rate (FDR) (Genovese et al., 2002) at a significance level of *p* < 0.05. Importantly, the ALE algorithm has been formulated to limit the inter-subject and inter-laboratory variability typical of neuroimaging studies. This algorithm estimates the spatial uncertainty of each focus and takes into account the possible differences among studies, as to avoid that single studies may drive the results.

We subdivided the cingulate cortex into areas with homogeneous co-activations by employing two kinds of cluster analysis (Cauda et al., 2010; Frades and Matthiesen, 2010): Hierarchical and k-means clustering and using as input the results of ALE analysis (MACM). Hierarchical clustering groups data over a variety of scales by creating a cluster tree; trees represent multilevel hierarchies where clusters at one level are joined as clusters at the next level. Importantly, hierarchical clustering does not require a priori impositions on the number of clusters and by creating the dendrogram it allows the visualization of the hierarchy within the data. The advantage of the hierarchical clustering is that it can handle different forms of similarity or distance. A distance matrix is the only requirement for hierarchical clustering. Furthermore, hierarchical clustering allows an embedded flexibility regarding the level of granularity; that is, the extent to which an entity is divided in smaller parts. We used the hierarchical clustering as we were interested in visualizing the hierarchical structure of the data. To verify the goodness of the hierarchical clustering we used the cophenetic distance. This measure describes how well the cluster tree reflects the data for different distance measures and allows to verify the consistency of each link. This method, together with the visual inspection of the dendrogram and the reordered distance matrix, represents a way to find the optimal number of clusters of the data (Ward, 1963). The matrix was composed of rows, representing the blocks formed by the quad tree algorithm, and columns representing the probability of activation obtained by the ALE analysis of each voxel in the brain. The data matrix was used to create the distance matrix. There are different criteria to evaluate the distances between clusters in the hierarchical clustering literature. In this case, we employed the Ward method that uses an analysis of variance approach (Ward, 1963). Subsequently, we employed the k-mean clustering to assess the results using as input the number of clusters obtained from the techniques described above.

Unlike hierarchical clustering, k-means operates on actual observations and creates a single level of clusters. K-means is a partition method in which objects are classified as belonging to one or k groups with k chosen a priori. The cluster membership is determined by calculating the centroid for each group and assigning each object to the group with the closest centroid. This approach minimizes the overall dispersion within cluster by operating an iterative reallocation of cluster members. Advantages of this methods are its time and space complexity and its order-independent properties. Order independency means that k-means generates the same partition of the data irrespective of the order in which patterns are presented.

The results of the k-means clustering were further verified using the average silhouette values (Rousseeuw, 1987). To calculate the hemisphere prevalence, for each cluster we overimposed the clusterization results of the left and the right cingulate cortex. Areas pertaining to the left cingulate cortex were colored in green, areas pertaining to the right cingulate cortex were colored in orange. Areas of overlap of the clusterization results of both sides were colored in red.

### Density analysis

We observed that voxels in the cingulate cortex were unequally activated by tasks. That is, the activation of some voxels was more frequent and some regions of the cingulate cortex were found to be characterized by a wider number of active voxels. In order to substantiate this observation, we performed a density analysis of the active foci in the cingulate cortex. This was done in order to obtain a deterministic method to calculate the density of foci. We decided not to use ALE for this aim because it modifies the probability kernel including information such as the number of subjects. In this way results are not exactly a measure of density of foci. For density, we intended the number of foci per unit of area. In order to analytically calculate the number of foci per unit per area, we used the Voronoi tessellation algorithm (Klein, 1989). A Voronoi tessellation is a decomposition of metric space by distances between sets of points. The Voronoi algorithm tessellates a surface into polygons in such a way that the area of each polygon is inversely proportional to the density of points (foci) in that area.

### Network analysis

It has been suggested that some portions of the cingulate cortex may act as hubs interconnecting different networks (Leech et al., 2011). Graph analysis techniques allow the investigation of how complex brain networks relate to each other (Bullmore and Sporns, 2009), therefore resulting particularly suitable for inspecting the presence of hub areas. Graph and network analyses build graphs of elements in such a way that the position of an element in the graph reflects its relationship with neighboring elements.

We reordered the distance matrix so as to place more edges closer to the diagonal. Reordering was performed using a routine of the Brain Connectivity Toolbox that minimizes the cost function of the matrix (Rubinov and Sporns, 2010). Then, using the data in the reordered distance matrix, we constructed a network and we optimally represented the results employing a force-directed algorithm: the Fruchterman-Reingold method (Fruchterman and Reingold, 1991). In this algorithm, the nodes are represented by steel rings and the edges are springs between them. The attractive force is analogous to the spring force and the repulsive force is analogous to the electrical force. The basic idea is to minimize the energy of the system by moving the nodes and changing the forces between them. A threshold was applied to the resulting image so that only the circles (blocks) with highest network connectivity (first quartile) were represented as color-filled.

## Experiments 2–4

We also performed three additional experiments on healthy volunteers.

### Participants

We scanned two groups of healthy volunteers. The first group was composed of 10 right-handed adults (five females) (mean age = 22 ± 1.4) who participated in the resting state experiment (experiment 2) and in the experiment in which emotional faces were shown (experiment 3). The second group was composed of 17 healthy right-handed volunteers (eight females, mean age 28 ± 4.2) who participated in the experiment in which painful stimuli were applied to the hands (experiment 3). All of the participants were free of neurological or psychiatric disorders, not taking medications known to alter brain activity, and with no history of drug or alcohol abuse. We obtained the written informed consent of each subject, in accordance with the Declaration of Helsinki. The studies were approved by the institutional committee on ethical use of human subjects at the University of Turin.

### Tasks and acquisition

#### Experiment 2—resting state

The first group performed a 6-minute resting state task. During the resting state scan, participants were asked to relax, to not fall asleep and to not think of anything in particular while they were being scanned.

#### Experiment 3—faces expressing emotions

The same 10 participants took part in an experiment in which faces expressing different emotions were shown to them while lying in the scanner. The stimuli consisted of faces showing anger, disgust, fear, happiness, sadness, and neutral expressions. The faces were taken from Biehl et al. (1997). A total of four Caucasian actors (two males and two females) showing both emotional and neutral expressions were used for a total of 24 images (six conditions × four actors). The task scans consisted of four runs of a slow event-related design. Each run consisted of 24 stimuli, four for each emotional and neutral condition, presented in random order. The subjects were instructed to look passively at the faces.

#### Experiment 4—painful stimuli

The second group participated in a task in which painful mechanical stimuli were delivered to the hands. Painful mechanical stimuli were delivered with a 256 mN pinprick probe which activates the high-threshold mechanoreceptors (Baumgartner et al., 2010). During the four runs of this slow event-related design, the participants received a total of 48 stimuli on the left and right hand (no more than three consecutive stimuli on the same hand). They were instructed to relax, pay attention to the stimuli and report a subjective rating of intensity at the end of each block.

#### Data acquisition

Data acquisition was performed on a 1.5 Tesla INTERA™ scanner (Philips Medical Systems) with a SENSE high-field, high resolution (MRIDC) head coil that was optimized for functional imaging. The functional T2^*^-weighted images were acquired using echoplanar (EPI) sequences, with a repetition time (TR) of 2000 ms, an echo time (TE) of 50 ms, and a 90° flip angle. The acquisition matrix was 64 × 64, and the field of view (FoV) 200 mm. A total of 200 volumes were acquired; each volume consisted of 19 axial slices, parallel to the anterior-posterior (AC-PC) commissure line and covering the whole brain; slice thickness was 4.5 mm with a 0.5 mm gap. Two scans were added at the beginning of the functional scanning and the data were discarded to reach a steady-state magnetization before acquiring the experimental data.

In the same session, a set of three-dimensional high-resolution T_1_-weighted structural images was acquired for each participant. This data-set was acquired using a Fast Field Echo (FFE) sequence, with a TR of 25 ms, ultra-short TE, and a 30° flip angle. The acquisition matrix was 256 × 256, and the field of view (FoV) 256 mm. The set consisted of 160 contiguous sagittal images covering the whole brain. In-plane resolution was 1 × 1 mm and slice thickness 1 mm (1 × 1 × 1 mm voxels).

### Analysis

The data were pre-processed using slice scan time correction, 3D motion correction, spatial smoothing (4 mm FWHM) and temporal filters (linear trend removal and band pass filter of 0.008–0.08 Hz).

After pre-processing, voxels belonging to the cingulate cortex were submitted to a voxel-wise unsupervised fuzzy c-mean clustering technique (Cauda et al., 2010, 2013). This data-driven method decomposes the original fMRI time series into a predefined number of spatiotemporal modes, which include a spatial map and an associated cluster centroid time course. The extent to which a voxel belongs to a cluster is defined by the similarity (as measured, e.g., by correlation) of its time course to the cluster centroid. We used this clusterization approach as it has been previously optimized for resting state and MACM (Cauda et al., 2010, 2011b).

We used two clustering approaches to compare the complexity of clustering of the MaC data to that of the experimental tasks. First, we imposed the same number of clusters obtained for the MaC to the in-house acquired datasets. Second, we calculated the number of clusters of the experimental data without imposing the results of the MaC.

### Validation of clustering procedures

We used the upper tail rule developed by Mojena to validate the number of clusters (Mojena, 1977). Statistical stopping rules for clustering methods allow selecting the “best” number of clusters in the data. Stopping rules define explicitly what is meant by a significant change in the clustering criterion. That is, these rules help define when a further partition is not necessary. The method developed by Mojena (1977) uses the relative sizes of the fusion level in the hierarchy. The algorithm selects the partition associated to the first level in the cluster number sequence which satisfies the following condition:

Zj + 1 > m + ksz

where *m* is the mean of the fusion level of the previous fusion level, *sz* is the standard deviation of those values, and *k* is the standard deviate. The knee of the curve of the cluster sequence is an indicator of the right number of clusters. In simple words, this method takes the distance matrix and calculates the types of aggregation by calculating mean and standard deviation of the number of nodes as a function of the imposed clusters. Then the knee is calculated as a second derivative of the curve. The peak that is obtained represents the “optimal” number of clusters according to the stopping criterion.

## Results

### Database search

The BrainMap query retrieved 1240 papers involving 24,540 subjects and a total of 1851 foci (see Table S1).

### MACM clustering

**Figure 1:**
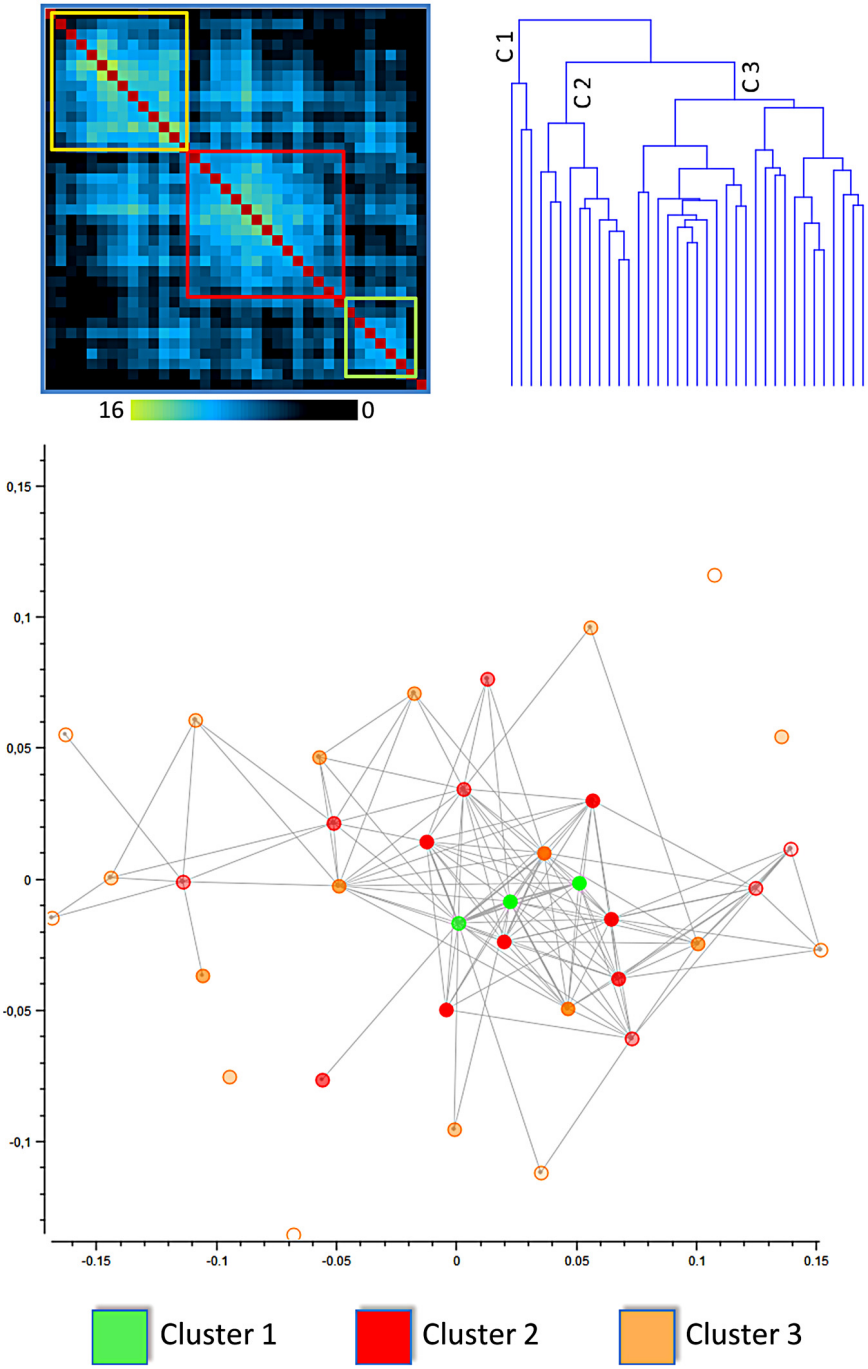
**Results of the clusterization of the cingulate cortex: distance matrix and multidimensional scaling (see also Figure 6 for network analysis)**. The upper left panel shows the results of the distance matrix. The upper right panel shows the dendrogram of the hierarchical clusterization, one of the algorithms used. The lower panel shows the Multidimensional scaling of the network derived from the distance between blocks of the cingulate cortex. The points represent blocks and are coded with a color indicating the cluster to which they belong. In this image the distance between points represents the Euclidean distance between the MACM maps of each block.

**Figure 2:**
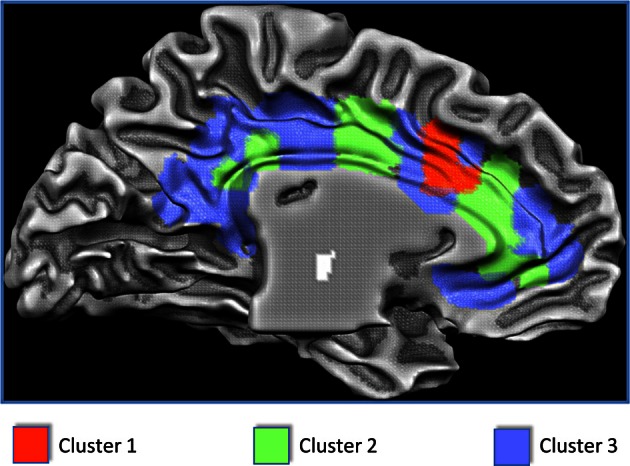
**Results of the clusterization of the cingulate cortex and anatomical location of the clusters**.

The results of the dendrogram obtained from the hierarchical clustering suggested that the cingulate cortex can be optimally parcellated into three clusters (see Figures 1, 2). The same result was confirmed by the silhouette plot. The silhouette plot is a measure of how close each point in one cluster is to points in the neighboring clusters. This method allows to understand of how well-separated the resulting clusters are.

We used the k-means method to cluster the MACM blocks into the three clusters as previously identified. To minimize the risk of inconsistent results obtained for the initial random placement of starting points, the k-means was computed 256 times (Nanetti et al., 2009). The same three clusters were identified all 256 times.

**Figure 3:**
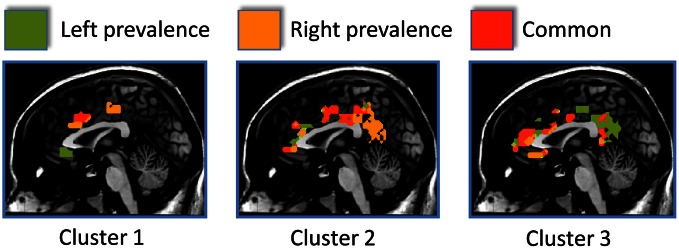
**The side differences for each cluster are shown here**. Areas showing a left prevalence are coded in green, areas showing a right prevalence are coded in orange, areas equally lateralized are coded in red.

The results of the MaC evidenced that Cluster 1 covers 7% of the cingulate surface and is located in the dorsal anterior cingulate cortex (dACC). Cluster 2 covers 30% of the total cingulate surface, being predominantly located in the intermediate part of the cingulate cortex but also presenting two small locations in the anterior and posterior cingulate cortex. Cluster 3 covers 63% of the cingulate surface, encompassing the posterior but also the anterior parts of the cingulate cortex, and also presenting a small location in the midcingulate area (Figures 2, 3).

**Figure 4:**
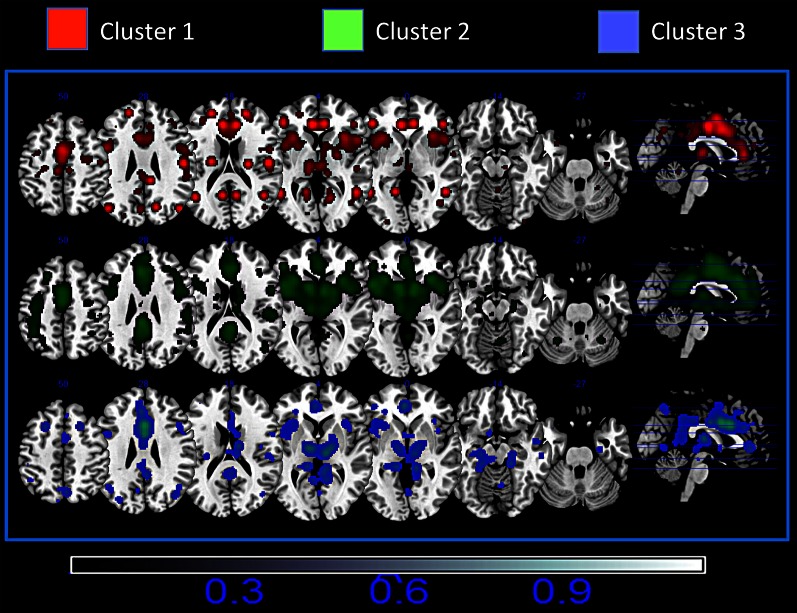
**Mean Meta-analytic connectivity of the three networks**. ALE maps were computed at an FDR-corrected threshold of *p* < 0.05; minimum cluster dimension *k* > 100 mm^3^ and visualized using MRIcron (http://www.cabiatl.com/mricro/mricron/index.html).

Figure 4 shows the mean MACM connectivity of the three clusters. Our results indicate that cluster 1 is functionally connected to the parietal cortices (the inferior parietal lobe BA 40, the superior parietal lobe BA 7), frontal cortices (superior and middle frontal gyri BA 9, 10) and to some sensory (primary somatosensory cortices BA 2, 3) and motor (primary and premotor areas BA 4 and 6) areas. In addition, this cluster shows connections to the temporal lobe (superior and middle temporal gyri BA 21, 22, 39) and is slightly left lateralized in the anterior cingulate cortex and slightly right lateralized in the posterior cingulate cortex. Cluster 2 is co-active with the inferior, middle and superior frontal gyri (BA 45, 9, and 10), the parietal cortex (BA 7, 40, 43), and the temporal lobe (BA 37, 39). Moreover this cluster presents a strong co-activation with subcortical structures such as the thalamus, the red nucleus, and the caudate. This cluster is also characterized by a right-prevalence of activations in the posterior cingulate cortex. Cluster 3 is characterized by a more frontal and limbic functional connectivity which includes the superior and middle frontal gyri (BA 9 and 10) and hippocampal and parahippocampal structures. All three clusters are extensively co-active with other portions of the cingulate cortex and with the insula (see Figure 4 and Tables S2–S4).

**Figure 5:**
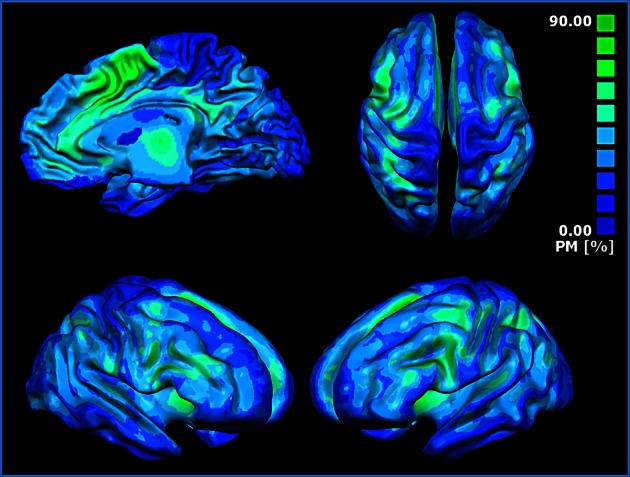
**Probabilistic map showing the superposition of the MACM of all blocks**. The probabilistic map shows the probability of overlap between each block-related MACM map. The probability map is calculated by summing the voxel value of each block-related MACM map and dividing this value by the number of blocks. Single network maps before the creation of the probability maps were thresholded at *p* < 0.05, minimum cluster size *k* > 100 mm^3^. Regions in blue are characterized by a low probability of overlap, whereas green regions present a high probability of overlap.

The probabilistic map (Figure 5) shows that the highest overlap between all the block-related MACM maps is in the anterior insula, dorsal cingulate cortex, dorsolateral prefrontal cortices, sensorimotor, precuneal, and posterior parietal cortices.

The lower part of Figure 1 represents the network derived from the distance between blocks of the cingulate cortex. The points represent blocks and are coded with a color indicating the cluster to which they belong. In this image the distance between points represents the Euclidean distance between the MACM maps of each block. The network representation is optimized using multidimensional scaling. Points belonging to cluster 1 are centrally located and surrounded by points belonging to clusters 2 and 3. This method rearranges multidimensional entities in a 2D space such that the distance represents the similarity. In this way, similar entities are closer in space whereas dissimilar entities are placed apart.

**Figure 6:**
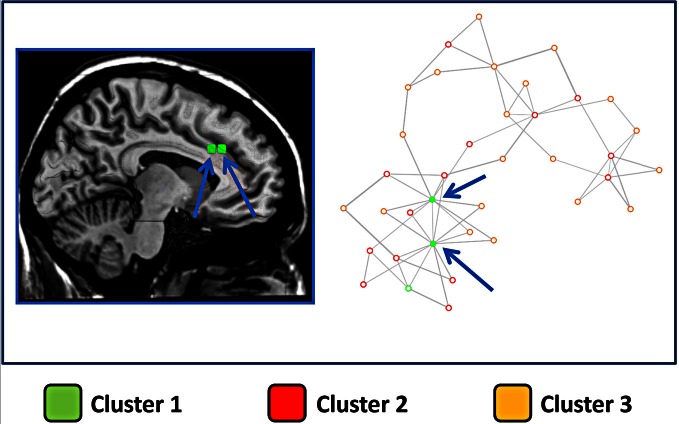
**Network derived from the distance between blocks of the cingulate cortex. Right panel:** The points represent blocks and are coded with a color indicating the cluster to which they belong. In this image the distance between points represents the Euclidean distance between the MACM maps of each block. The network representation is optimized using a force-directed layout algorithm (Fruchterman–Reingold). Arrows indicate the two blocks with the highest number of connections. **Left panel**: the two blocks with the highest number of connections are graphically represented over a sliced standard brain surface.

The same finding was confirmed by the results presented in Figure 6, which shows the network representation of the Euclidean distances between blocks. Here the graphical network representation is optimized with the Fruchterman–Reingold method. The color-filled circles display blocks with the highest degree of connectivity. This analysis supports the idea that blocks in cluster 1 are hub areas.

A representation of which behavioral classes activate each cluster can be found in Figure S1.

### Density of foci

**Figure 7:**
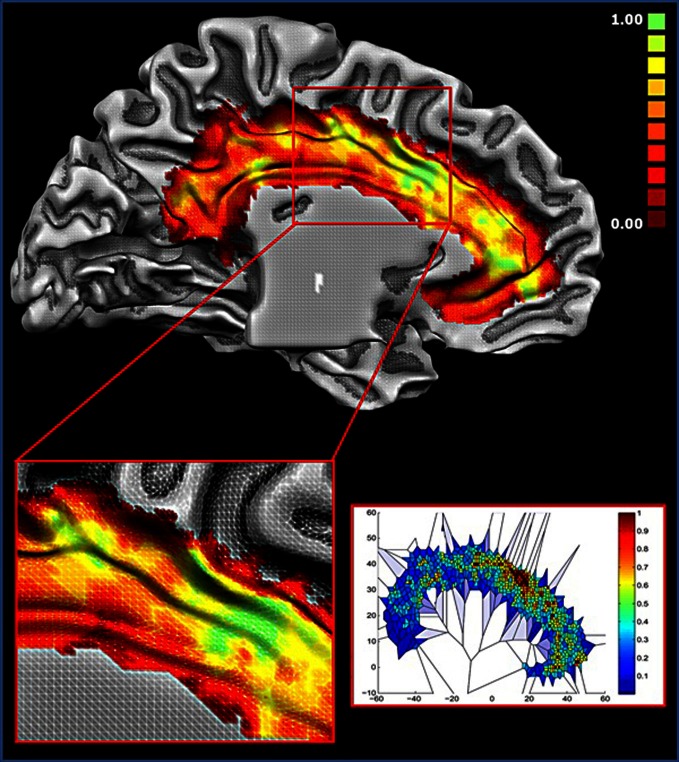
**Density of foci. Upper panel**: colors from red to green represent increased foci density. **Lower right panel**: Voronoi tessellation of the cingulate cortex; colors from blue to red are inversely proportional to the Voronoi polygon area (i.e., Proportional to the density). All values are normalized.

Figure 7 displays the results of the Voronoi tessellation. The dACC showed the highest density of foci. Notably, this area was found to partially overlap with the location of cluster 1. Other portions in the anterior cingulate cortex and in the cingulate motor zone showed a high density of foci. The two blocks showing the highest number of functional connections (see Figure 6) were found to be part of cluster 1 and were located in the dorsal cingulate cortex in the area with the highest density of foci. Together, these results indicate that such area exerts a pivotal role as a hub.

Figure S2 describes the behavioral related density of foci.

### Experiments 2–4

Images of the task-evoked activations and of the resting state protocol are shown in the supplementary material (Figures S3–S5).

**Figure 8:**
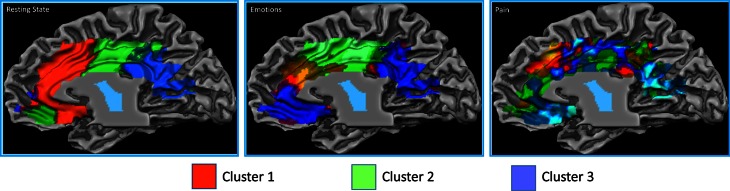
**Control experiments**. Connectivity-based parcellation of the cingulate cortex in the three datasets. Probabilistic maps for functional connectivity defined clusters. The color scheme represents the probability of overlapping brain areas in each voxel across all the subjects. Maps are projected on an inflated 3D brain surface with the BrainVoyager QX surface tool. This parcellation was obtained by imposing the same number of clusters obtained for the parcellation of the MaC data.

The resting state parcellation shows a tripartite subdivision of the cingulate cortex with an anterior, a mid and a posterior cluster. In contrast, the parcellations based on the functional connectivity of the cingulate cortex when involved in the elaboration of emotional faces are less sharp and more complex (Figure 8). The parcellation based on responses to painful stimuli goes further in the direction of the results of the emotions paradigm: The clusters were found to be intermixed in a complex and blurred pattern of connectivity. Areas clearly connected with one network in the resting state showed, in contrast, an intermixed pattern of connectivity during tasks. That is, areas previously found to be characterized by homogeneous functional connectivity (e.g., cluster containing only voxels belonging to that cluster) are found to contain voxels also belonging to other clusters.

**Figure 9:**
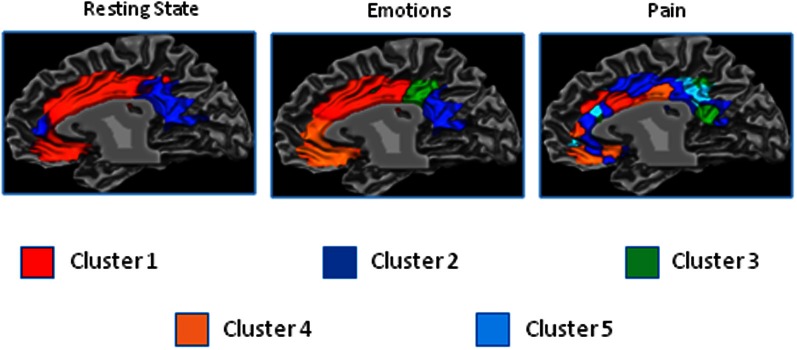
**Control experiments**. Connectivity-based parcellation of the cingulate cortex in the three datasets. Probabilistic maps for functional connectivity defined clusters. The color scheme represents the probability of overlapping brain areas in each voxel across all the subjects. Maps are projected on an inflated 3D brain surface with the BrainVoyager QX surface tool. This parcellation was obtained by parcellating the data without imposing an a priori partition.

The results of the parcellation of the cingulate cortex based on the activations elicited by experimental tasks and obtained without imposing a priori the number of clusters, revealed a pattern of increasing complexity from the resting state data (2 clusters) to the emotion (3 clusters) and pain (5 clusters) paradigms (See Figure 9 and Figure S6 for the results of the stopping criterion).

## Discussion

This study was designed to investigate the pattern of clusterization of the cingulate cortex during the broadest range of tasks possible. We performed a meta-analytic study using a meta-analytic tool recently developed by our group: the MaC (Cauda et al., 2012). We further performed three additional fMRI experiments on healthy volunteers. In this way we could observe possible modifications of the parcellation of the cingulate cortex during the widest number of active tasks, during specific tasks and during resting state. Our main finding was that the cingulate cortex changes its pattern of co-activations depending on the level of task complexity. Indeed, the complexity of the pattern of parcellation increased and changed from the resting state experiment, to the two task-based experiments and the meta-analytic study. Importantly, we considered the task complexity not as reflecting a greater cognitive challenge, but rather as reflecting the kind of task (resting state-no task vs. active tasks) and the number of tasks (e.g., responses to painful stimulation vs. a general active mode, that is whenever a task is performed-meta-analytic study).

### MaC clustering: clustering MACM results

Voxel-based meta-analyses have provided a fundamental contribution to the building of new insights into brain functions. In a previous study, we found that an ROI based parcellation of the cingulate cortex produced three clusters (Torta and Cauda, 2011). Such results were confirmed by those of the present study which indicate that using MaC, the cingulate cortex can be efficiently divided into three clusters (see Figure 1). These three clusters have different dimensions and cover a different percentage of the cingulate parenchyma. Although each cluster is characterized by specific co-activations (Corbetta et al., 1991, 2008; Corbetta and Shulman, 2002; Seeley et al., 2007; Beckmann et al., 2009; Menon and Uddin, 2010; Alexander and Brown, 2011), all three have extensive co-activations within the cingulate cortex and the insula (Cauda et al., 2011b, 2012) and share an “attentional” pattern of co-activation (Craig, 2003, 2009; Medford and Critchley, 2010; Cauda et al., 2011b; Torta and Cauda, 2011). This overlap of common co-activations is maximal in the dACC and in the insula, as evidenced by the results of the probabilistic map (Figure 5). These findings support the view that the cingulate cortex and the insula form a saliency network devoted to the integration of information coming from the internal (e.g., homeostasis) and the external (e.g., sensory) environments (e.g., sensory inputs) (Vincent et al., 2008). In addition, this insular-cingulate system is thought to be in charge providing a stable “set maintenance” over the execution of tasks (Dosenbach et al., 2006). Moreover, we found that all clusters are strongly locally interconnected as evidenced by the presence of activity of the dACC and of the insula in the MACM profiles of the three clusters.

One possible criticism to our approach is that it may be considered not completely data driven. However, to obtain a voxel-wise analysis we first needed to aggregate a certain number of voxels to reach a minimum amount of foci. In this sense, our clustering cannot be entirely considered as voxelwise. Our method does not rely on pre-determined regions of interest but operates a local reduction of the resolution in a inverse relation to the density of foci. Other alternative solutions have been proposed (Bzdok et al., 2012). For instance, Bzdok and colleagues (2012) have obtained a partial voxel-wise clusterization by searching foci in the adjacency of a voxel, spreading the search to reach a minimum number of foci and then projecting the connectivity profile of these foci to the voxel.

### Hubs of the cingulate cortex

The overlap of activations in the dACC, insula and thalamus (see Figure 5) suggests that such brain regions act as hubs through which homeostatic and sensory information is conveyed. This interpretation is also supported by the results of the Voronoi tessellation that pointed to the dACC as the area with the greatest density of foci. Indeed, it is highly likely that those areas having the greatest density of foci also have a greater number of connections. Further confirmation of the interpretation of the dACC as a hub area comes from the results of the distance matrix analysis (Figure 1, lower panel) and of the network analysis. Indeed, the findings of the graph analysis show that, in multidimensional scaling of MACM-based profiles, blocks of cluster 1 are close to each other and placed in a central position. These results indicated that such blocks have a similar and homogenous connectivity pattern. Moreover, they also suggest that cluster 1 may exert a pivotal role in regulating the activity of the other two clusters. A pivotal role of the cingulo-insular network in the regulation of the activity of other networks was suggested by Sridharan and colleagues (2008). These authors proposed that the cingulo-insular network plays a major role in switching between brain networks such as the central-executive network and the default-mode network (Sridharan et al., 2008). Interestingly, hypo-functionality of the dACC has been related to reduced awareness of the self. This would suggest that damage to hub areas may result in a failure to integrate stimuli coming from the external world into a coherent representation of the self (Amanzio et al., 2011).

### Parcellation of the cingulate cortex when at rest and when involved in active tasks

Whether the cingulate cortex has a segregated functional organization or not remains a matter of debate. Devinsky et al. (1995) and Bush et al. (2000) have reviewed evidence from neuroimaging, neurophysiological and anatomical data supporting the view that each part of the cingulate cortex is specialized in a family of cognitive operations, with the anterior cingulate more devoted to affective elaboration and the midcingulate to cognitive tasks. Previous studies, investigating the parcellation of the cingulate cortex on the basis of its connectivity (anatomical, Beckmann et al., 2009 or functional Yu et al., 2011) have upheld this view. A partial support for these results came also from a previous study performed by our group (Torta and Cauda, 2011). However, our present findings open up the possibility of a substantial divergence between the parcellation of the cingulate cortex when at rest and when involved in active tasks. Differences were also found depending on the kind and on the number of active tasks under exam. This result suggests that, when involved in active tasks, the cingulate cortex reconfigures its patterns of co-activation. In this view, the cingulate cortex may modify its connectivity from a resting state to a “working state”: We, propose that such a modification may be related to the kind of task executed and consequently to how subpopulations of neurons modify their own specific time courses to begin to cooperate with different neuronal groups (see also Leech et al., 2011). This implies that such subdivisions are probably related to the kind of task and that, depending on the kind of task that is included in the analysis, they may change. In this sense, it may be argued that not even the MACM results allow a generalization of a “functional clustering” of the cingulate cortex, as also in BrainMap some tasks are more represented than others. The results of the experiments seem to support this view. However, two elements should be considered. First, these observations do not hamper the main results of our study, namely that the cingulate cortex reconfigures to a more complex parcellation when involved in active tasks. Second, although some tasks are indeed more represented than others in BrainMap, it is also true that some cognitive functions such as attention are common across a great variety of tasks and thus more likely to be always recruited. Indeed, a growing body of literature indicates that the intrinsic functional connectivity of a brain region is modulated in function of a task, both during and after task execution (Liu et al., 1999; Friston and Buchel, 2000; Lowe et al., 2000; Hampson et al., 2002; Jiang et al., 2004; Peltier et al., 2005; Waites et al., 2005; Fransson, 2006; Hasson et al., 2009; Lewis et al., 2009; Tambini et al., 2010) and that the functional connectivity varies adaptively with a local efficiency that is higher locally and lower globally (Wang et al., 2012). Here, we did not directly measure functional connectivity, but used MACM, which is meant to assess the consistency of co-activations. However, it has been shown that MACM and resting state functional connectivity may lead to similar results (Cauda et al., 2011a). It is difficult to explain how such co-activations of brain areas across different paradigms and studies may emerge in absence of any functional connectivity between these areas [for a discussion see Koski and Paus (2000), Postuma and Dagher (2006), Laird et al. (2009b), Smith et al. (2009)]. Functional co-activations may be thus interpreted as forms of functional connectivity (Koski and Paus, 2000; Postuma and Dagher, 2006; Laird et al., 2009b; Smith et al., 2009). Furthermore, the mapping of functional connectivity via coordinate-based meta-analysis has been validated by comparing the results of MACM to resting-state connectivity (Smith et al., 2009). Both approaches produced very consistent results.

Another important question regards the possibility that complex “interdigitated” results may be related to under or over-decompositions in the clustering step. This issue specifically applies to clusterization in the control experiments. We used two different approaches to investigate if the cingulate cortex reconfigures its pattern of connectivity from resting state to “working state.” First, we kept the number of clusters of the in-house acquired datasets equal to the number of clusters calculated for the meta-analytic data in order to have a one-by-one comparison between clusters. The logic behind this choice was to compare how the pattern changes across different levels of task complexity. Indeed, the evidence of greater functional complexity of a given region can be reflected either by an increase in the number of clusters (some of which probably very small) or by a more complex and interdigitated clusterization (as in our case). In addition, it should be considered that, as evidenced by Figure S2, single behavioral categories of stimuli do not activate contiguous cingulate areas but wide regions of interdigitated (sparse) blobs. This further supports the view that “sparseness” predominates in brain connectivity (Daubechies et al., 2009). However, as a second approach, in order to validate the results obtained with the first, we performed a parcellation of the resting state and task results, without imposing to the experimental datasets the same number of clusters as in the MaC. The results of this second analysis confirmed what we proposed with the previous approach, namely that from resting state to experimental tasks, there is an increase in the number of identified clusters (from 2 of the resting state to 5 of the pain task).

### Limitations of the study; neurobiological meaning of the results and controversies

Our results seem at odds with previous neurobiological evidence showing many additional partitions in the cingulate cortex (Bush et al., 2000; Vogt, 2009). However, some elements should be borne in mind when interpreting our findings. First, different parcellation schemes may co-exist in associative areas such as the insula (Cauda and Vercelli, 2012; Kelly et al., 2012). This could be explained by the intrinsically hierarchical nature of brain networks, as disclosed by neuroimaging studies (Doucet et al., 2011; Power et al., 2011; Yeo et al., 2011). Second, functional properties of these regions do not necessarily correspond to the anatomical subdivisions. For instance, it has been proposed that different tasks and stimuli may activate the very same region of the cingulate cortex as this area may be devoted to elaborate some common characteristics of the tasks (Vogt, 2005). Third, the aim of our paper was not to provide compelling evidence to the parcellation of the cingulate cortex when during tasks. This is difficult, as in meta-analytic studies, many papers on different behavioral domains are pooled together. Thus, it may well be that different behavioral domains lead to different co-activations and thus to different parcellations. This is what we show with the control experiments. In addition, it has been demonstrated that clusterization of brain networks likely produces sparse rather than segregated results (Daubechies et al., 2009). All these elements may explain why it is possible to obtain a clusterization of the cingulate cortex that is different from previously suggested ones. In addition, it has been shown that individual anatomical differences may produce slightly diverging results when parcellating the cingulate cortex (Beckmann et al., 2009). We could not control for these factors in the meta-analytic study, thus our results might have been affected also by this. Importantly, the main objective of our study was to show that when the cingulate cortex is studied during tasks, the co-activations of its subparts diverge in a way that, when clustered together, is different from what we have known on the cingulate cortex so far. Finally, previous studies using meta-analytic procedures have already shown that our understanding of the cingulate cortex during tasks remains behind its anatomical understanding. Our clustering is based mainly on co-activations (MACM procedure), thus in this sense it is possible that a part of the cingulate cortex, as for example the anterior cingulate cortex, is connected with others as they are functionally linked at a network level, although the two components remain anatomically segregated.

In conclusion, we have shown that the cingulate cortex changes its pattern of co-activations depending on the level of task complexity. In addition, clusters resulting from a parcellation of the cingulate cortex during tasks, rather than being strongly functionally segregated, are likely to reflect the activity of hub areas densely interconnected with local and whole-brain networks. This opens up the possibility that areas that appear to be active for a wide range of active tasks are differentially recruited by each of them and echo the activity of other networks in the brain (Leech et al., 2011).

### Conflict of interest statement

The authors declare that the research was conducted in the absence of any commercial or financial relationships that could be construed as a potential conflict of interest.

## Abbreviations

MaC: Meta-analytic clustering
fMRI: Functional magnetic resonance imaging
ROI: Region of interest
MACM: Meta-analytic connectivity modeling
ALE: Activation likelihood estimation.
