# Supplementary material for: Parcellation of the cingulate cortex at rest and during tasks: a meta-analytic clustering and experimental study
Source: Front Hum Neurosci. 2013 Jun 14;7:275. doi: 10.3389/fnhum.2013.00275 (PMC3682391; doi:10.3389/fnhum.2013.00275)
Supplement: Supplementary file 1 [file DataSheet1.DOCX]

SUPPLEMENTARY MATERIAL

This file contains the list of papers included in the study (Table S1), the description of clusters 1, 2 and 3 (Tables S2-S4) and two supplementary figures (S1 and S2)

Table S1: List of the papers included in the study

| YEAR | FIRST AUTHOR | JOURNAL |
| --- | --- | --- |
| 1988 | Petersen | Nature |
| 1989 | Petersen | Journal of Cognitive Neuroscience |
| 1990 | Pardo | Proceedings of the National Academy of Sciences |
| 1991 | Colebatch | Journal of Neurophysiology |
| 1991 | Colebatch | Journal of Physiology |
| 1991 | Seitz | European Journal of Neuroscience |
| 1992 | Drevets | Journal of Neuroscience |
| 1992 | Grafton | Journal of Neuroscience |
| 1992 | Seitz | European Journal of Neuroscience |
| 1992 | Sergent | Cerebral Cortex |
| 1993 | Bench | Neuropsychologia |
| 1993 | Grafton | Experimental Brain Research |
| 1993 | Paus | Journal of Neurophysiology |
| 1993 | Ramsay | Journal of Physiology |
| 1994 | Anderson | Brain |
| 1994 | Coghill | Journal of Neuroscience |
| 1994 | Decety | Nature |
| 1994 | Demonet | Brain |
| 1994 | George | Human Brain Mapping |
| 1994 | Grasby | Brain |
| 1994 | Jenkins | Journal of Neuroscience |
| 1995 | Andreasen | American Journal of Psychiatry |
| 1995 | Berman | Neuropsychologia |
| 1995 | Bonda | Proceedings of the National Academy of Sciences |
| 1995 | Bookheimer | Human Brain Mapping |
| 1995 | Dettmers | Journal of Neurophysiology |
| 1995 | Fiez | Journal of Cognitive Neuroscience |
| 1995 | Friston | NeuroImage |
| 1995 | George | American Journal of Psychiatry |
| 1995 | Ghatan | NeuroImage |
| 1995 | O'Driscoll | Proceedings of the National Academy of Sciences |
| 1995 | O'Sullivan | Neuroreport |
| 1995 | Partiot | Neuroreport |
| 1995 | Stephan | Journal of Neurophysiology |
| 1996 | Andreasen | Proceedings of the National Academy of Sciences |
| 1996 | Awh | Psychological Science |
| 1996 | Blaxton | Journal of Neuroscience |
| 1996 | Casey | Journal of Neurophysiology |
| 1996 | Fiez | Journal of Neuroscience |
| 1996 | Fink | Journal of Applied Physiology |
| 1996 | Fletcher | Brain |
| 1996 | Fox | Nature |
| 1996 | Fukuyama | Neuroreport |
| 1996 | Gemar | Depression |
| 1996 | George | Biological Psychiatry |
| 1996 | Grafton | Experimental Brain Research |
| 1996 | Ito | Journal of Nuclear Medicine |
| 1996 | Kapur | Journal of Cognitive Neuroscience |
| 1996 | Kawashima | Brain Research |
| 1996 | Larsson | Neuroreport |
| 1996 | Maquet | Nature |
| 1996 | Martin | Nature |
| 1996 | Morris | Nature |
| 1996 | Nagahama | Brain |
| 1996 | Nyberg | Proceedings of the National Academy of Sciences |
| 1996 | Petit | Journal of Neuroscience |
| 1996 | Price | Brain |
| 1996 | Price | Proceedings of the Royal Society of London. Series B. Biological Sciences |
| 1996 | Schumacher | NeuroImage |
| 1996 | Smith | Cerebral Cortex |
| 1996 | Sweeney | Journal of Neurophysiology |
| 1996 | Warburton | Brain |
| 1997 | Adler | Anesthesia and Analgesia |
| 1997 | Alivisatos | Neuropsychologia |
| 1997 | Baker | Psychological Medicine |
| 1997 | Beauregard | Journal of Cognitive Neuroscience |
| 1997 | Blok | Brain |
| 1997 | Brammer | Magnetic Resonance Imaging |
| 1997 | Burton | Cerebral Cortex |
| 1997 | Cohen | Nature |
| 1997 | Doricchi | Experimental Brain Research |
| 1997 | Elliott | Neuropsychologia |
| 1997 | Fox | Neuroreport |
| 1997 | Ganguli | Biological Psychiatry |
| 1997 | Grafton | NeuroImage |
| 1997 | Henke | Hippocampus |
| 1997 | Imaizumi | Neuroreport |
| 1997 | Jennings | NeuroImage |
| 1997 | Jonides | Journal of Cognitive Neuroscience |
| 1997 | Jueptner | NeuroImage |
| 1997 | Kertzman | Experimental Brain Research |
| 1997 | Lane | American Journal of Psychiatry |
| 1997 | Lane | Neuroreport |
| 1997 | Law | Acta Physiologica Scandinavica |
| 1997 | Mayberg | Neuroreport |
| 1997 | Nobre | Brain |
| 1997 | Paradiso | American Journal of Psychiatry |
| 1997 | Phillips | Nature |
| 1997 | Rao | Journal of Neuroscience |
| 1997 | Rao | Neuroreport |
| 1997 | Rauch | Human Brain Mapping |
| 1997 | Reiman | American Journal of Psychiatry |
| 1997 | Rumsey | Brain |
| 1997 | Sadato | Journal of Neuroscience |
| 1997 | Samuel | Brain |
| 1997 | Spence | Brain |
| 1997 | Svensson | Journal of Neurophysiology |
| 1997 | Taylor | NeuroImage |
| 1997 | Vandenberghe | Journal of Neuroscience |
| 1997 | Winstein | Journal of Neurophysiology |
| 1997 | Xu | Neuroreport |
| 1998 | Beauregard | Neuroreport |
| 1998 | Blok | Brain |
| 1998 | Boecker | Journal of Neurophysiology |
| 1998 | Buchel | Neuron |
| 1998 | Buckner | NeuroImage |
| 1998 | Canli | Neuroreport |
| 1998 | Carlson | Cerebral Cortex |
| 1998 | Casey | NeuroImage |
| 1998 | Catalan | Brain |
| 1998 | Corbetta | Neuron |
| 1998 | Curtis | American Journal of Psychiatry |
| 1998 | Derbyshire | Experimental Brain Research |
| 1998 | Ellermann | Journal of Magnetic Resonance |
| 1998 | Elliott | Psychological Medicine |
| 1998 | Fredrikson | Psychophysiology |
| 1998 | Ghatan | NeuroImage |
| 1998 | Goerres | Neuroreport |
| 1998 | Grady | Proceedings of the National Academy of Sciences |
| 1998 | Griffiths | Nature Neuroscience |
| 1998 | Halsband | Behavioural Brain Research |
| 1998 | Heckers | Nature Neuroscience |
| 1998 | Kawashima | Experimental Brain Research |
| 1998 | Kelley | Neuron |
| 1998 | Kohler | Neuropsychologia |
| 1998 | Konishi | European Journal of Neuroscience |
| 1998 | Konishi | Nature Neuroscience |
| 1998 | LaBar | Neuron |
| 1998 | Lumer | Science |
| 1998 | Luna | Cerebral Cortex |
| 1998 | Mattay | Psychiatry Research |
| 1998 | Morris | Brain |
| 1998 | Owen | Proceedings of the National Academy of Sciences |
| 1998 | Paulson | Pain |
| 1998 | Perani | Brain |
| 1998 | Phillips | Psychiatry Research |
| 1998 | Ragland | Neuropsychology |
| 1998 | Rajah | NeuroImage |
| 1998 | Sadato | Brain |
| 1998 | Schlosser | Journal of Neurology, Neurosurgery, and Psychiatry |
| 1998 | Svensson | European Journal of Pain |
| 1998 | Taylor | NeuroImage |
| 1998 | Tzourio-Mazoyer | NeuroImage |
| 1998 | Wagner | Neuroreport |
| 1998 | Whalen | Biological Psychiatry |
| 1998 | Zald | Brain |
| 1998 | van | Journal of Neurophysiology |
| 1999 | Berman | Human Brain Mapping |
| 1999 | Binkofski | European Journal of Neuroscience |
| 1999 | Blair | Brain |
| 1999 | Blood | Nature Neuroscience |
| 1999 | Brown | Journal of the International Neuropsychological Society |
| 1999 | Catalan | Brain |
| 1999 | Chochon | Journal of Cognitive Neuroscience |
| 1999 | Coghill | Journal of Neurophysiology |
| 1999 | Corfield | Journal of Applied Physiology |
| 1999 | Crespo-Facorro | American Journal of Psychiatry |
| 1999 | Curtis | Schizophrenia Research |
| 1999 | Elliott | Neuropsychologia |
| 1999 | Fiez | Neuron |
| 1999 | Francis | Neuroreport |
| 1999 | Garavan | Proceedings of the National Academy of Sciences |
| 1999 | Gelnar | NeuroImage |
| 1999 | Gitelman | Brain |
| 1999 | Hagoort | Journal of Cognitive Neuroscience |
| 1999 | Halpern | Cerebral Cortex |
| 1999 | Hamdy | American Journal of Physiology |
| 1999 | Henke | Proceedings of the National Academy of Sciences |
| 1999 | Henson | Journal of Neuroscience |
| 1999 | Isenberg | Proceedings of the National Academy of Sciences |
| 1999 | Joliot | NeuroImage |
| 1999 | Kang | NeuroImage |
| 1999 | Kawashima | Neuroscience |
| 1999 | Kim | NeuroImage |
| 1999 | Kimbrell | Biological Psychiatry |
| 1999 | Klein | Neuroreport |
| 1999 | LaBar | NeuroImage |
| 1999 | Mayberg | American Journal of Psychiatry |
| 1999 | Moore | Brain |
| 1999 | Moore | NeuroImage |
| 1999 | Mottaghy | Experimental Brain Research |
| 1999 | Naito | Journal of Neuroscience |
| 1999 | Omori | Neuroscience Research |
| 1999 | Paradiso | American Journal of Psychiatry |
| 1999 | Perry | Neuroreport |
| 1999 | Petit | Journal of Neurophysiology |
| 1999 | Peyron | Brain |
| 1999 | Poldrack | NeuroImage |
| 1999 | Poldrack | Neuropsychology |
| 1999 | Rauch | Psychiatry Research |
| 1999 | Rogers | Journal of Neuroscience |
| 1999 | Rosen | Journal of Cognitive Neuroscience |
| 1999 | Rypma | NeuroImage |
| 1999 | Sathian | Journal of Cognitive Neuroscience |
| 1999 | Shen | Human Brain Mapping |
| 1999 | Shin | American Journal of Psychiatry |
| 1999 | Shulman | Journal of Neuroscience |
| 1999 | Sturm | Neuropsychologia |
| 1999 | Tataranni | Proceedings of the National Academy of Sciences |
| 1999 | Tatsumi | NeuroImage |
| 1999 | Teasdale | American Journal of Psychiatry |
| 1999 | Wu | American Journal of Psychiatry |
| 1999 | Zald | Annals of Neurology |
| 2000 | Arrington | Journal of Cognitive Neuroscience |
| 2000 | Audenaert | European Journal of Nuclear Medicine |
| 2000 | Aziz | Journal of Neuroscience |
| 2000 | Banich | Journal of Cognitive Neuroscience |
| 2000 | Barnes | Neuropsychologia |
| 2000 | Bartels | Neuroreport |
| 2000 | Binkofski | Human Brain Mapping |
| 2000 | Buchanan | Cognitive Brain Research |
| 2000 | Callicott | Cerebral Cortex |
| 2000 | Carlsson | Journal of Cognitive Neuroscience |
| 2000 | Carter | Proceedings of the National Academy of Sciences |
| 2000 | Cohen | Brain |
| 2000 | Connolly | Journal of Neurophysiology |
| 2000 | Critchley | Human Brain Mapping |
| 2000 | Dove | Cognitive Brain Research |
| 2000 | Ehrsson | European Journal of Neuroscience |
| 2000 | Ehrsson | Journal of Neurophysiology |
| 2000 | Elliott | Journal of Neuroscience |
| 2000 | Etard | Neuroreport |
| 2000 | Fischer | Behavioral Neuroscience |
| 2000 | Garavan | Cerebral Cortex |
| 2000 | Gerardin | Cerebral Cortex |
| 2000 | Hopfinger | Nature Neuroscience |
| 2000 | Houde | Journal of Cognitive Neuroscience |
| 2000 | Hui | Human Brain Mapping |
| 2000 | Iidaka | Journal of Cognitive Neuroscience |
| 2000 | Ingham | Brain and Language |
| 2000 | Jancke | Cognitive Brain Research |
| 2000 | Jancke | Neuropsychologia |
| 2000 | Johansen-Berg | Neuroreport |
| 2000 | Kawashima | Journal of Neurophysiology |
| 2000 | Kiehl | Psychophysiology |
| 2000 | Kim | American Journal of Psychiatry |
| 2000 | Kircher | Neuroreport |
| 2000 | Knutson | NeuroImage |
| 2000 | Kogure | Journal of Nuclear Medicine |
| 2000 | Kohler | Acta Psychologica |
| 2000 | Kuperberg | Journal of Cognitive Neuroscience |
| 2000 | Laureys | Brain |
| 2000 | Le | NeuroImage |
| 2000 | Lee | Annals of Neurology |
| 2000 | Leung | Cerebral Cortex |
| 2000 | Leveroni | Journal of Neuroscience |
| 2000 | Liberzon | Neuropsychopharmacology |
| 2000 | Liotti | Biological Psychiatry |
| 2000 | Lutz | Neuroreport |
| 2000 | MacDonald | Science |
| 2000 | Marois | Neuron |
| 2000 | Martinkauppi | Cerebral Cortex |
| 2000 | Mayberg | Biological Psychiatry |
| 2000 | Mechelli | Journal of Cognitive Neuroscience |
| 2000 | Mencl | Microscopy Research and Technique |
| 2000 | Naito | Journal of Neurophysiology |
| 2000 | Nobre | NeuroImage |
| 2000 | Nour | Brain |
| 2000 | O'Leary | Neuroreport |
| 2000 | Pesenti | Journal of Cognitive Neuroscience |
| 2000 | Petrovic | Pain |
| 2000 | Pietrini | American Journal of Psychiatry |
| 2000 | Pihlajamaki | Annals of Neurology |
| 2000 | Riecker | Neuroreport |
| 2000 | Rogers | Journal of Cognitive Neuroscience |
| 2000 | Rosen | Neurology |
| 2000 | Rowe | Science |
| 2000 | Sabatini | Brain |
| 2000 | Sakurai | Cognitive Brain Research |
| 2000 | Savic | Neuron |
| 2000 | Seitz | Experimental Brain Research |
| 2000 | Siebner | Neurology |
| 2000 | Sobel | Journal of Neurophysiology |
| 2000 | Sohn | Proceedings of the National Academy of Sciences |
| 2000 | Stanescu-Cosson | Brain |
| 2000 | Stern | NeuroImage |
| 2000 | Stevens | Magnetic Resonance Imaging |
| 2000 | Sugiura | NeuroImage |
| 2000 | Tan | Human Brain Mapping |
| 2000 | Taylor | Neuropsychologia |
| 2000 | Tracey | Neuroscience Letters |
| 2000 | Weder | Human Brain Mapping |
| 2000 | Xiong | NeuroImage |
| 2000 | Yatham | Archives of General Psychiatry |
| 2001 | Ackermann | Neuroreport |
| 2001 | Athwal | Brain |
| 2001 | Banich | Progress in Brain Research |
| 2001 | Barch | Archives of General Psychiatry |
| 2001 | Barch | Cerebral Cortex |
| 2001 | Berns | Journal of Neuroscience |
| 2001 | Blood | Proceedings of the National Academy of Sciences |
| 2001 | Bocher | NeuroImage |
| 2001 | Bodegard | Neuron |
| 2001 | Bonte | Journal of Nuclear Medicine |
| 2001 | Brannan | Proceedings of the National Academy of Sciences |
| 2001 | Braver | Cerebral Cortex |
| 2001 | Bunge | Brain |
| 2001 | Calautti | Stroke |
| 2001 | Calhoun | NeuroImage |
| 2001 | Carter | American Journal of Psychiatry |
| 2001 | Casey | Journal of Neurophysiology |
| 2001 | Chee | NeuroImage |
| 2001 | Coghill | Journal of Neurophysiology |
| 2001 | Crespo-Facorro | Human Brain Mapping |
| 2001 | Crespo-Facorro | Journal of American Medical Association |
| 2001 | Critchley | Neuron |
| 2001 | Curtis | Journal of Affective Disorders |
| 2001 | Damasio | NeuroImage |
| 2001 | Daselaar | NeuroImage |
| 2001 | Dassonville | NeuroImage |
| 2001 | Dehaene | Nature Neuroscience |
| 2001 | Druzgal | Cognitive Brain Research |
| 2001 | Druzgal | Neuron |
| 2001 | Duzel | Human Brain Mapping |
| 2001 | Ehrsson | Journal of Neurophysiology |
| 2001 | Fletcher | Nature Neuroscience |
| 2001 | Goel | Neuropsychologia |
| 2001 | Grady | NeuroImage |
| 2001 | Grosbras | Cerebral Cortex |
| 2001 | Gusnard | Proceedings of the National Academy of Sciences |
| 2001 | Heide | European Journal of Neuroscience |
| 2001 | Herath | Cerebral Cortex |
| 2001 | Hobday | Brain |
| 2001 | Indovina | Experimental Brain Research |
| 2001 | Just | NeuroImage |
| 2001 | Kennedy | American Journal of Psychiatry |
| 2001 | Kiehl | Schizophrenia Research |
| 2001 | Kimmig | Experimental Brain Research |
| 2001 | Knutson | Journal of Neuroscience |
| 2001 | Knutson | Neuroreport |
| 2001 | Kuo | Neuroreport |
| 2001 | Lotze | NeuroImage |
| 2001 | Maclin | Neuroreport |
| 2001 | Martin-Soelch | European Journal of Neuroscience |
| 2001 | Martin-Solch | Experimental Brain Research |
| 2001 | Martin | Journal of Neurophysiology |
| 2001 | Mayer | Neuroreport |
| 2001 | Mazoyer | Brain Research Bulletin |
| 2001 | Menon | Human Brain Mapping |
| 2001 | Menon | NeuroImage |
| 2001 | Merriam | NeuroImage |
| 2001 | Milham | Cognitive Brain Research |
| 2001 | Monchi | Journal of Neuroscience |
| 2001 | Muley | NeuroImage |
| 2001 | Nakamura | Neuropsychologia |
| 2001 | Nunn | Nature Neuroscience |
| 2001 | O'Doherty | Journal of Neurophysiology |
| 2001 | O'Doherty | Nature Neuroscience |
| 2001 | Palmer | NeuroImage |
| 2001 | Perlstein | American Journal of Psychiatry |
| 2001 | Pesenti | Nature Neuroscience |
| 2001 | Pfefferbaum | NeuroImage |
| 2001 | Pinel | NeuroImage |
| 2001 | Pizzagalli | American Journal of Psychiatry |
| 2001 | Pochon | Cerebral Cortex |
| 2001 | Poellinger | NeuroImage |
| 2001 | Poldrack | Brain |
| 2001 | Poldrack | Journal of Cognitive Neuroscience |
| 2001 | Ragland | American Journal of Psychiatry |
| 2001 | Rama | NeuroImage |
| 2001 | Rao | Nature Neuroscience |
| 2001 | Rossell | Neuropsychologia |
| 2001 | Rubia | NeuroImage |
| 2001 | Rubia | Schizophrenia Research |
| 2001 | Rypma | Psychology and Aging |
| 2001 | Savage | Brain |
| 2001 | Savic | Neuron |
| 2001 | Schiltz | Cortex |
| 2001 | Shergill | Psychological Medicine |
| 2001 | Shulman | Proceedings of the National Academy of Sciences |
| 2001 | Sperling | Human Brain Mapping |
| 2001 | Sugiura | NeuroImage |
| 2001 | Tan | NeuroImage |
| 2001 | Tan | Neuroreport |
| 2001 | Temple | Neuroreport |
| 2001 | Ullsperger | NeuroImage |
| 2001 | Vandenberghe | NeuroImage |
| 2001 | Videbech | Psychological Medicine |
| 2001 | Wagner | Neuron |
| 2001 | Xu | Cerebral Cortex |
| 2001 | Zago | NeuroImage |
| 2001 | Zysset | Neuroscience Letters |
| 2001 | de | Human Brain Mapping |
| 2001 | van | NeuroImage |
| 2002 | Adleman | NeuroImage |
| 2002 | Alexander | American Journal of Psychiatry |
| 2002 | Arnow | Brain |
| 2002 | Audenaert | Nuclear Medicine Communications |
| 2002 | Balsamo | Archives of Neurology |
| 2002 | Bantick | Brain |
| 2002 | Boecker | NeuroImage |
| 2002 | Booth | Human Brain Mapping |
| 2002 | Booth | NeuroImage |
| 2002 | Bornhovd | Brain |
| 2002 | Brass | Cerebral Cortex |
| 2002 | Bunge | NeuroImage |
| 2002 | Cabeza | NeuroImage |
| 2002 | Canli | Proceedings of the National Academy of Sciences |
| 2002 | Cansino | Cerebral Cortex |
| 2002 | Chao | Cerebral Cortex |
| 2002 | Cohen | Brain |
| 2002 | Cooke | Human Brain Mapping |
| 2002 | Derbyshire | Journal of Pain |
| 2002 | Dreher | NeuroImage |
| 2002 | Dreher | Proceedings of the National Academy of Sciences |
| 2002 | Dupont | Surgical and Radiologic Anatomy |
| 2002 | Durston | NeuroImage |
| 2002 | Elliott | Archives of General Psychiatry |
| 2002 | Erk | Neuroreport |
| 2002 | Fincham | Proceedings of the National Academy of Sciences |
| 2002 | Fu | NeuroImage |
| 2002 | Gagnon | Brain |
| 2002 | Garavan | NeuroImage |
| 2002 | Gauthier | Neuron |
| 2002 | Grafton | Experimental Brain Research |
| 2002 | Grezes | Neuropsychologia |
| 2002 | Gurd | Brain |
| 2002 | Halsband | Behavioural Brain Research |
| 2002 | Haslinger | Cognitive Brain Research |
| 2002 | Hautzel | Neuroscience Letters |
| 2002 | Henson | NeuroImage |
| 2002 | Hutchinson | NeuroImage |
| 2002 | Iwase | NeuroImage |
| 2002 | Johnson | Brain |
| 2002 | Johnson | NeuroImage |
| 2002 | Jordan | Neuropsychologia |
| 2002 | Karama | Human Brain Mapping |
| 2002 | Kelley | Journal of Cognitive Neuroscience |
| 2002 | Kim | NeuroImage |
| 2002 | Kircher | Neuropsychologia |
| 2002 | Konishi | Proceedings of the National Academy of Sciences |
| 2002 | Kroger | Cerebral Cortex |
| 2002 | Langheim | NeuroImage |
| 2002 | Langleben | NeuroImage |
| 2002 | Lanius | Biological Psychiatry |
| 2002 | Lee | Human Brain Mapping |
| 2002 | Leung | Journal of Cognitive Neuroscience |
| 2002 | Luke | Human Brain Mapping |
| 2002 | Luks | NeuroImage |
| 2002 | Mazard | Journal of Cognitive Neuroscience |
| 2002 | Mead | Journal of the International Neuropsychological Society |
| 2002 | Mellet | Cerebral Cortex |
| 2002 | Meyer | Human Brain Mapping |
| 2002 | Milham | Brain and Cognition |
| 2002 | Miller | Journal of Cognitive Neuroscience |
| 2002 | Nathaniel-James | NeuroImage |
| 2002 | Noppeney | NeuroImage |
| 2002 | Nunneley | Journal of Applied Physiology |
| 2002 | O'Leary | Neuropsychopharmacology |
| 2002 | Ochsner | Journal of Cognitive Neuroscience |
| 2002 | Ortuno | NeuroImage |
| 2002 | Pastor | Journal of Neuroscience |
| 2002 | Pessoa | Neuron |
| 2002 | Pessoa | Proceedings of the National Academy of Sciences |
| 2002 | Peterson | Cognitive Brain Research |
| 2002 | Petrovic | NeuroImage |
| 2002 | Petrovic | Science |
| 2002 | Pochon | Proceedings of the National Academy of Sciences |
| 2002 | Ragland | Neuropsychology |
| 2002 | Ravnkilde | Journal of Clinical and Experimental Neuropsychology |
| 2002 | Rotte | Stereotactic and Functional Neurosurgery |
| 2002 | Rushworth | Journal of Neurophysiology |
| 2002 | Schmidt | Neuropsychologia |
| 2002 | Seidler | Science |
| 2002 | Sevostianov | International Journal of Neuroscience |
| 2002 | Siebner | Journal of Neuroscience |
| 2002 | Simon | Neuron |
| 2002 | Skaf | Journal of Affective Disorders |
| 2002 | Smith | British Journal of Psychiatry |
| 2002 | Smith | Neurology |
| 2002 | Staudt | NeuroImage |
| 2002 | Stephan | NeuroImage |
| 2002 | Suchan | Behavioural Brain Research |
| 2002 | Suzuki | NeuroImage |
| 2002 | Szameitat | Journal of Cognitive Neuroscience |
| 2002 | Tanabe | NeuroImage |
| 2002 | Turkeltaub | NeuroImage |
| 2002 | Umetsu | NeuroImage |
| 2002 | Veit | Neuroscience Letters |
| 2002 | Vingerhoets | NeuroImage |
| 2002 | Watanabe | NeuroImage |
| 2002 | Wildgruber | NeuroImage |
| 2002 | Winterer | NeuroImage |
| 2002 | Wykes | British Journal of Psychiatry |
| 2002 | Yucel | American Journal of Psychiatry |
| 2003 | Abel | Neuroreport |
| 2003 | Adcock | NeuroImage |
| 2003 | Akitsuki | NeuroImage |
| 2003 | Andreasen | Human Brain Mapping |
| 2003 | Astafiev | Journal of Neuroscience |
| 2003 | Beauchamp | NeuroImage |
| 2003 | Binder | Journal of Cognitive Neuroscience |
| 2003 | Braver | Neuron |
| 2003 | Cabeza | Journal of Cognitive Neuroscience |
| 2003 | Calarge | American Journal of Psychiatry |
| 2003 | Calvert | Journal of Cognitive Neuroscience |
| 2003 | Carr | Proceedings of the National Academy of Sciences |
| 2003 | Coghill | Proceedings of the National Academy of Sciences |
| 2003 | Cohen | Cerebral Cortex |
| 2003 | Daselaar | Neurobiology of Aging |
| 2003 | Daumann | Neuroreport |
| 2003 | De | Journal of Fluency Disorders |
| 2003 | De | Journal of Neurolinguistics |
| 2003 | De | Journal of Neurophysiology |
| 2003 | Delazer | Cognitive Brain Research |
| 2003 | Desmond | NeuroImage |
| 2003 | Dilger | Neuroscience Letters |
| 2003 | Dobbins | Neuropsychologia |
| 2003 | Dreher | Cerebral Cortex |
| 2003 | Durston | Biological Psychiatry |
| 2003 | Durston | NeuroImage |
| 2003 | Ehrsson | Journal of Neurophysiology |
| 2003 | Fan | NeuroImage |
| 2003 | Ferrandez | NeuroImage |
| 2003 | Fossati | American Journal of Psychiatry |
| 2003 | Gandour | Human Brain Mapping |
| 2003 | Ganis | Cerebral Cortex |
| 2003 | Garavan | NeuroImage |
| 2003 | Gerardin | Cerebral Cortex |
| 2003 | Giesbrecht | NeuroImage |
| 2003 | Gould | NeuroImage |
| 2003 | Gruber | NeuroImage |
| 2003 | Gusnard | Proceedings of the National Academy of Sciences |
| 2003 | Hanakawa | Journal of Neurophysiology |
| 2003 | Hariri | Biological Psychiatry |
| 2003 | Honey | Psychological Medicine |
| 2003 | Horn | Neuropsychologia |
| 2003 | Jeffries | Neuroreport |
| 2003 | Jensen | Neuron |
| 2003 | Killgore | NeuroImage |
| 2003 | Kim | American Journal of Psychiatry |
| 2003 | Kirsch | NeuroImage |
| 2003 | Knecht | NeuroImage |
| 2003 | Knutson | NeuroImage |
| 2003 | Konishi | Journal of Neuroscience |
| 2003 | Kotz | Brain and Language |
| 2003 | Kringelbach | Cerebral Cortex |
| 2003 | Kubicki | NeuroImage |
| 2003 | Kuhtz-Buschbeck | European Journal of Neuroscience |
| 2003 | Kumari | Biological Psychiatry |
| 2003 | Kumari | Psychiatry Research |
| 2003 | Kuo | NeuroImage |
| 2003 | Lanius | Biological Psychiatry |
| 2003 | Lazeron | Journal of the Neurological Sciences |
| 2003 | Linden | NeuroImage |
| 2003 | Lorenz | Brain |
| 2003 | MacDonald | Journal of Abnormal Psychology |
| 2003 | Markowitsch | Cortex |
| 2003 | Martin-Soelch | European Journal of Neuroscience |
| 2003 | Matsuo | Cognitive Brain Research |
| 2003 | McKay | Journal of Applied Physiology |
| 2003 | Milham | Cognitive Brain Research |
| 2003 | Mitchell | Neuropsychologia |
| 2003 | Mitterschiffthaler | Neuroreport |
| 2003 | Mort | NeuroImage |
| 2003 | Mostofsky | Cognitive Brain Research |
| 2003 | Muller | American Journal of Psychiatry |
| 2003 | Nemoto | Neuroreport |
| 2003 | Neumann | Journal of Fluency Disorders |
| 2003 | O'Doherty | Neuron |
| 2003 | O'Doherty | Neuropsychologia |
| 2003 | Okada | Neuropsychobiology |
| 2003 | Onozuka | Journal of Dental Research |
| 2003 | Pelletier | Neuroreport |
| 2003 | Perani | Human Brain Mapping |
| 2003 | Perlstein | Biological Psychiatry |
| 2003 | Piefke | Brain |
| 2003 | Pihlajamaki | Hippocampus |
| 2003 | Potenza | American Journal of Psychiatry |
| 2003 | Preibisch | NeuroImage |
| 2003 | Ramnani | Cerebral Cortex |
| 2003 | Ranganath | Neuropsychologia |
| 2003 | Remy | NeuroImage |
| 2003 | Riecker | NeuroImage |
| 2003 | Rolls | Cerebral Cortex |
| 2003 | Rolls | European Journal of Neuroscience |
| 2003 | Rubia | NeuroImage |
| 2003 | Ruge | Journal of Magnetic Resonance Imaging |
| 2003 | Seifritz | Biological Psychiatry |
| 2003 | Shapira | Biological Psychiatry |
| 2003 | Siok | Human Brain Mapping |
| 2003 | Small | Neuron |
| 2003 | Sonty | Annals of Neurology |
| 2003 | Specht | NeuroImage |
| 2003 | Specht | Neuroscience Letters |
| 2003 | Speer | Biological Psychiatry |
| 2003 | Sperling | Journal of Neurology, Neurosurgery, and Psychiatry |
| 2003 | Sperling | NeuroImage |
| 2003 | Strigo | Journal of Neurophysiology |
| 2003 | Suzuki | Dysphagia |
| 2003 | Swainson | Journal of Cognitive Neuroscience |
| 2003 | Sylvester | Neuropsychologia |
| 2003 | Tan | Human Brain Mapping |
| 2003 | Taniwaki | Journal of Neuroscience |
| 2003 | Tapert | Archives of General Psychiatry |
| 2003 | Taylor | NeuroImage |
| 2003 | Ueda | Neuroreport |
| 2003 | Ullsperger | Journal of Neuroscience |
| 2003 | Vanlancker-Sidtis | Brain and Language |
| 2003 | Vingerhoets | NeuroImage |
| 2003 | Walter | Schizophrenia Research |
| 2003 | Weiss | Neuroscience Letters |
| 2003 | Weiss | Psychiatry Research |
| 2003 | Wicker | Brain Research Reviews |
| 2003 | Wicker | Neuron |
| 2003 | Wild | Psychiatry Research |
| 2003 | Winston | NeuroImage |
| 2003 | Wood | Journal of Cognitive Neuroscience |
| 2003 | Wraga | Brain and Cognition |
| 2003 | Wrase | Neuroscience Letters |
| 2003 | Yoo | Neuroreport |
| 2003 | de | European Journal of Neuroscience |
| 2003 | de | Journal of Neurophysiology |
| 2003 | van | Hearing Research |
| 2004 | Abrahams | Brain |
| 2004 | Aron | Journal of Neurophysiology |
| 2004 | Bellgrove | Neuropsychologia |
| 2004 | Bestmann | European Journal of Neuroscience |
| 2004 | Bischoff-Grethe | Cognitive Brain Research |
| 2004 | Bjork | Journal of Neuroscience |
| 2004 | Booth | Journal of Cognitive Neuroscience |
| 2004 | Brass | Journal of Cognitive Neuroscience |
| 2004 | Brown | Cognitive Brain Research |
| 2004 | Brown | Neuroreport |
| 2004 | Cairo | Cognitive Brain Research |
| 2004 | Calhoun | Human Brain Mapping |
| 2004 | Cato | Journal of Cognitive Neuroscience |
| 2004 | Chang | Archives of General Psychiatry |
| 2004 | Chen | NeuroImage |
| 2004 | Cools | Journal of Neuroscience |
| 2004 | Coull | Science |
| 2004 | Critchley | Nature Neuroscience |
| 2004 | Cunningham | Journal of Cognitive Neuroscience |
| 2004 | Daselaar | NeuroImage |
| 2004 | Decety | NeuroImage |
| 2004 | Elliott | Biological Psychiatry |
| 2004 | Elliott | NeuroImage |
| 2004 | Ernst | Neuropsychologia |
| 2004 | Fahim | Brain and Cognition |
| 2004 | Fassbender | Cognitive Brain Research |
| 2004 | Ferrarelli | Brain Research Bulletin |
| 2004 | Fiehler | European Journal of Neuroscience |
| 2004 | Fitzgerald | Neuroscience Letters |
| 2004 | Fu | Archives of General Psychiatry |
| 2004 | Giesecke | Arthritis & Rheumatism |
| 2004 | Grezes | Journal of Neuroscience |
| 2004 | Hall | Neuroreport |
| 2004 | Haruno | Journal of Neuroscience |
| 2004 | Haslinger | Human Brain Mapping |
| 2004 | Herz | Neuropsychologia |
| 2004 | Hugdahl | American Journal of Psychiatry |
| 2004 | Ibinson | Anesthesiology |
| 2004 | Ino | Brain Research Bulletin |
| 2004 | Ischebeck | Journal of Cognitive Neuroscience |
| 2004 | Jackson | NeuroImage |
| 2004 | Jansma | Schizophrenia Research |
| 2004 | Kanayama | Psychopharmacology |
| 2004 | Kawabata | Journal of Neurophysiology |
| 2004 | Kelly | European Journal of Neuroscience |
| 2004 | Kerr | Neuroreport |
| 2004 | Kikyo | NeuroImage |
| 2004 | Kindermann | Schizophrenia Research |
| 2004 | Kircher | NeuroImage |
| 2004 | Knutson | Neuron |
| 2004 | Kobayashi | NeuroImage |
| 2004 | Konen | NeuroImage |
| 2004 | Kozel | Behavioral Neuroscience |
| 2004 | Kozel | Journal of Neuropsychiatry and Clinical Neuroscience |
| 2004 | Kuo | NeuroImage |
| 2004 | Landau | NeuroImage |
| 2004 | Lanius | American Journal of Psychiatry |
| 2004 | Lee | Cognitive Behavioral Neurology |
| 2004 | Leibenluft | Biological Psychiatry |
| 2004 | Lennox | Psychological Medicine |
| 2004 | Lerner | NeuroImage |
| 2004 | Levine | Journal of Cognitive Neuroscience |
| 2004 | Liu | NeuroImage |
| 2004 | Macrae | Cerebral Cortex |
| 2004 | Maguire | NeuroImage |
| 2004 | Mainero | NeuroImage |
| 2004 | Malhi | Bipolar Disorders |
| 2004 | Malhi | European Journal of Neuroscience |
| 2004 | Martin | Journal of Neurophysiology |
| 2004 | Mataix-Cols | Archives of General Psychiatry |
| 2004 | Mathews | Journal of Cognitive Neuroscience |
| 2004 | Matsuda | Psychiatry Research |
| 2004 | Monks | Bipolar Disorders |
| 2004 | Morris | NeuroImage |
| 2004 | Najib | American Journal of Psychiatry |
| 2004 | Numminen | NeuroImage |
| 2004 | O'Doherty | Science |
| 2004 | Ochsner | Journal of Cognitive Neuroscience |
| 2004 | Peelen | NeuroImage |
| 2004 | Pelchat | NeuroImage |
| 2004 | Phelps | Neuron |
| 2004 | Phillips | NeuroImage |
| 2004 | Pierce | Brain |
| 2004 | Poeppel | Neuropsychologia |
| 2004 | Ragland | American Journal of Psychiatry |
| 2004 | Reyes | Hearing Research |
| 2004 | Rilling | Neuroreport |
| 2004 | Ruby | Journal of Cognitive Neuroscience |
| 2004 | Salgado-Pineda | NeuroImage |
| 2004 | Salomons | Journal of Neuroscience |
| 2004 | Schmitz | NeuroImage |
| 2004 | Seymour | Nature |
| 2004 | Sharot | Nature Neuroscience |
| 2004 | Singer | Science |
| 2004 | Siok | Nature |
| 2004 | Smith | Human Brain Mapping |
| 2004 | Steele | NeuroImage |
| 2004 | Thiel | NeuroImage |
| 2004 | Valet | Pain |
| 2004 | Watanabe | NeuroImage |
| 2004 | Weiss | Schizophrenia Research |
| 2004 | Xue | Neuroreport |
| 2004 | Zink | Neuron |
| 2004 | de | Journal of Neuroscience |
| 2004 | van | Cognitive Brain Research |
| 2005 | Achim | Journal of Cognitive Neuroscience |
| 2005 | Altshuler | Biological Psychiatry |
| 2005 | Amir | Biological Psychiatry |
| 2005 | Anand | Biological Psychiatry |
| 2005 | Aoki | Experimental Brain Research |
| 2005 | Aron | Journal of Neurophysiology |
| 2005 | Audoin | Human Brain Mapping |
| 2005 | Bedwell | International Journal of Neuroscience |
| 2005 | Bengtsson | European Journal of Neuroscience |
| 2005 | Bestmann | NeuroImage |
| 2005 | Bondi | Neurology |
| 2005 | Bonner-Jackson | Biological Psychiatry |
| 2005 | Breitenstein | NeuroImage |
| 2005 | Bristow | NeuroImage |
| 2005 | Caldwell | Behavioral Neuroscience |
| 2005 | Canli | Proceedings of the National Academy of Sciences |
| 2005 | Cannon | Archives of General Psychiatry |
| 2005 | Connolly | Journal of Neurophysiology |
| 2005 | Coricelli | Nature Neuroscience |
| 2005 | Cox | Journal of Neuroscience |
| 2005 | Creem-Regehr | Cognitive Brain Research |
| 2005 | Denslow | Biological Psychiatry |
| 2005 | Dresel | NeuroImage |
| 2005 | Ernst | NeuroImage |
| 2005 | Fenker | European Journal of Neuroscience |
| 2005 | Ferretti | NeuroImage |
| 2005 | Fitzgerald | Biological Psychiatry |
| 2005 | Floyer-Lea | Journal of Neurophysiology |
| 2005 | Ford | Journal of Neurophysiology |
| 2005 | Garraux | Journal of Neuroscience |
| 2005 | Goekoop | NeuroImage |
| 2005 | Goldin | NeuroImage |
| 2005 | Grandjean | Nature Neuroscience |
| 2005 | Griffiths | Journal of Urology |
| 2005 | Habel | NeuroImage |
| 2005 | Haller | Neuropsychologia |
| 2005 | Harris | Journal of Cerebral Blood Flow and Metabolism |
| 2005 | Harvey | NeuroImage |
| 2005 | Hennenlotter | NeuroImage |
| 2005 | Hirao | NeuroImage |
| 2005 | Holsen | NeuroImage |
| 2005 | Huettel | Journal of Neuroscience |
| 2005 | Hui | NeuroImage |
| 2005 | Ishii | European Journal of Nuclear Medicine and Molecular Imaging |
| 2005 | Jackson | NeuroImage |
| 2005 | Jeong | Psychiatry Research |
| 2005 | Keedwell | Biological Psychiatry |
| 2005 | Kerns | American Journal of Psychiatry |
| 2005 | Killgore | Developmental Psychobiology |
| 2005 | Kitada | NeuroImage |
| 2005 | Kozel | Biological Psychiatry |
| 2005 | Kuhnen | Neuron |
| 2005 | Kuhtz-Buschbeck | Journal of Urology |
| 2005 | Kulkami | European Journal of Neuroscience |
| 2005 | Lacourse | NeuroImage |
| 2005 | Langleben | Human Brain Mapping |
| 2005 | Lanius | Biological Psychiatry |
| 2005 | Laurens | Schizophrenia Research |
| 2005 | Liebenthal | Cerebral Cortex |
| 2005 | Malisza | Pediatric Research |
| 2005 | Maltby | NeuroImage |
| 2005 | Manoach | Schizophrenia Research |
| 2005 | Meister | Human Brain Mapping |
| 2005 | Meltzer | NeuroImage |
| 2005 | Mendrek | Psychological Medicine |
| 2005 | Milham | Human Brain Mapping |
| 2005 | Mo | Human Brain Mapping |
| 2005 | Mu | Sleep |
| 2005 | Nakao | Psychiatry Research |
| 2005 | Nieuwenhuis | European Journal of Neuroscience |
| 2005 | Nieuwenhuis | NeuroImage |
| 2005 | Nowak | Human Brain Mapping |
| 2005 | Nunez | NeuroImage |
| 2005 | O'Boyle | Cognitive Brain Research |
| 2005 | Ochsner | NeuroImage |
| 2005 | Ongur | Psychiatry Research |
| 2005 | Parsons | Human Brain Mapping |
| 2005 | Perico | Neuroscience Letters |
| 2005 | Phan | Biological Psychiatry |
| 2005 | Podzebenko | Journal of Cognitive Neuroscience |
| 2005 | Poldrack | Journal of Neuroscience |
| 2005 | Prince | Journal of Neuroscience |
| 2005 | Protopopescu | Proceedings of the National Academy of Sciences |
| 2005 | Puttemans | Journal of Neuroscience |
| 2005 | Ragland | American Journal of Psychiatry |
| 2005 | Rodriguez-Fornells | Journal of Cognitive Neuroscience |
| 2005 | Rounis | NeuroImage |
| 2005 | Schulz | Cerebral Cortex |
| 2005 | Seung | Neuroscience Research |
| 2005 | Specht | Neuroscience Letters |
| 2005 | Stark | Biological Psychology |
| 2005 | Tham | NeuroImage |
| 2005 | Tremblay | Archives of General Psychiatry |
| 2005 | Van | Proceedings of the National Academy of Sciences |
| 2005 | Vandekerckhove | Behavioural Neurology |
| 2005 | Vink | Human Brain Mapping |
| 2005 | Vitali | Brain and Language |
| 2005 | Volle | Cerebral Cortex |
| 2005 | Wildgruber | NeuroImage |
| 2005 | Woodruff | Neuropsychologia |
| 2005 | Yonelinas | Journal of Neuroscience |
| 2005 | Yoo | International Journal of Neuroscience |
| 2005 | van | Archives of General Psychiatry |
| 2006 | Abe | Cerebral Cortex |
| 2006 | Adcock | Neuron |
| 2006 | Addis | NeuroImage |
| 2006 | Allen | Psychopharmacology |
| 2006 | Aramaki | Cerebral Cortex |
| 2006 | Aron | Journal of Neuroscience |
| 2006 | Assaf | Biological Psychiatry |
| 2006 | Assaf | Psychiatry Research |
| 2006 | Baumgartner | Brain Research |
| 2006 | Bayless | Neuroscience Letters |
| 2006 | Beauregard | Neuroreport |
| 2006 | Becerra | Anesthesia and Analgesia |
| 2006 | Bermpohl | Human Brain Mapping |
| 2006 | Bingel | Pain |
| 2006 | Blair | Journal of Neuroscience |
| 2006 | Bohland | NeuroImage |
| 2006 | Borroni | Neurobiology of Aging |
| 2006 | Brambati | Brain Research |
| 2006 | Britton | NeuroImage |
| 2006 | Brown | Cerebral Cortex |
| 2006 | Brown | European Journal of Neuroscience |
| 2006 | Brown | NeuroImage |
| 2006 | Calhoun | Human Brain Mapping |
| 2006 | Callan | NeuroImage |
| 2006 | Camchong | Biological Psychiatry |
| 2006 | Carlsson | NeuroImage |
| 2006 | Chen | Biological Psychiatry |
| 2006 | Coan | Psychological Science |
| 2006 | Cunnington | NeuroImage |
| 2006 | Dapretto | Nature Neuroscience |
| 2006 | Daselaar | Cerebral Cortex |
| 2006 | Daselaar | Journal of Neurophysiology |
| 2006 | David | Journal of Cognitive Neuroscience |
| 2006 | Daw | Nature |
| 2006 | Deeley | British Journal of Psychiatry |
| 2006 | Denkova | Neuropsychologia |
| 2006 | Desseilles | NeuroImage |
| 2006 | Dolcos | Journal of Neuroscience |
| 2006 | Drobyshevsky | NeuroImage |
| 2006 | Evers | Psychopharmacology |
| 2006 | Farrell | Proceedings of the National Academy of Sciences |
| 2006 | Fliessbach | NeuroImage |
| 2006 | Fu | Cerebral Cortex |
| 2006 | Garrett | NeuroImage |
| 2006 | Gizewski | Experimental Brain Research |
| 2006 | Grimm | NeuroImage |
| 2006 | Grosbras | Cerebral Cortex |
| 2006 | Halsband | Journal of Physiology - Paris |
| 2006 | Hampton | Journal of Neuroscience |
| 2006 | Harenski | NeuroImage |
| 2006 | Hermann | Alcoholism: Clinical and Experimental Research |
| 2006 | Hirshorn | Neuropsychologia |
| 2006 | Hoeft | Journal of Neuroscience |
| 2006 | Ischebeck | NeuroImage |
| 2006 | Jackson | Neuropsychologia |
| 2006 | Jacobsen | NeuroImage |
| 2006 | Jager | Psychopharmacology |
| 2006 | Jahanshahi | Journal of Neuroscience |
| 2006 | Johnson | Biological Psychiatry |
| 2006 | Johnson | Neurobiology of Aging |
| 2006 | Kawachi | European Journal of Nuclear Medicine and Molecular Imaging |
| 2006 | Kensinger | Journal of Neuroscience |
| 2006 | Kerns | NeuroImage |
| 2006 | Kim | International Journal of Impotence Research |
| 2006 | Kim | PLoS Biology |
| 2006 | Kirsch | Neuroscience Letters |
| 2006 | Knutson | Social Neuroscience |
| 2006 | Kong | Human Brain Mapping |
| 2006 | Kumari | Schizophrenia Research |
| 2006 | Lee | Social Cognitive and Affective Neuroscience |
| 2006 | Lehericy | Cerebral Cortex |
| 2006 | Little | Brain and Cognition |
| 2006 | Maihofner | European Journal of Neuroscience |
| 2006 | Mathiak | Human Brain Mapping |
| 2006 | Mechelli | NeuroImage |
| 2006 | Meisenzahl | European Archives of Psychiatry and Clinical Neuroscience |
| 2006 | Meschyan | NeuroImage |
| 2006 | Mochizuki-Kawai | Brain Research |
| 2006 | Mohamed | Radiology |
| 2006 | Montaldi | Hippocampus |
| 2006 | Moriguchi | NeuroImage |
| 2006 | Mostofsky | Biological Psychiatry |
| 2006 | Moulier | NeuroImage |
| 2006 | Ongur | Archives of General Psychiatry |
| 2006 | Platek | Human Brain Mapping |
| 2006 | Ponseti | NeuroImage |
| 2006 | Ragland | Schizophrenia Research |
| 2006 | Rektor | Experimental Brain Research |
| 2006 | Remijnse | Archives of General Psychiatry |
| 2006 | Ricciardi | Neuroscience |
| 2006 | Riecker | NeuroImage |
| 2006 | Roth | Neuroreport |
| 2006 | Rubia | Human Brain Mapping |
| 2006 | Sacco | NeuroImage |
| 2006 | Saccuman | NeuroImage |
| 2006 | Saito | Neuroreport |
| 2006 | Schmahl | Archives of General Psychiatry |
| 2006 | Soros | NeuroImage |
| 2006 | Staresina | Journal of Neuroscience |
| 2006 | Tang | Journal of Cognitive Neuroscience |
| 2006 | Uher | Behavioural Brain Research |
| 2006 | Voets | Brain |
| 2006 | Vollm | NeuroImage |
| 2006 | Wagner | Biological Psychiatry |
| 2006 | Wittfoth | NeuroImage |
| 2006 | Yacubian | Journal of Neuroscience |
| 2006 | Yin | Journal of Nuclear Medicine |
| 2006 | Yokoyama | NeuroImage |
| 2006 | Yoon | Neuroscience Letters |
| 2006 | de | Human Brain Mapping |
| 2006 | de | Oral Surgery, Oral Medicine, Oral Pathology, Oral Radiology, and Endodontics |
| 2007 | Abe | Journal of Cognitive Neuroscience |
| 2007 | Abler | Journal of Psychiatric Research |
| 2007 | Abler | Psychopharmacology |
| 2007 | Addis | Neuropsychologia |
| 2007 | Ahrens | Brain and Language |
| 2007 | Altamura | Psychiatry Research |
| 2007 | Basho | Neuropsychologia |
| 2007 | Behrens | Nature Neuroscience |
| 2007 | Beneventi | Scandinavian Journal of Psychology |
| 2007 | Benuzzi | Brain Research Bulletin |
| 2007 | Bjork | Behavioural Brain Research |
| 2007 | Blair | NeuroImage |
| 2007 | Bremner | Journal of Affective Disorders |
| 2007 | Brown | Brain and Cognition |
| 2007 | Brown | Journal of Neurophysiology |
| 2007 | Butler | Neuroscience |
| 2007 | Carreiras | Journal of Cognitive Neuroscience |
| 2007 | Cheng | Current Biology |
| 2007 | Chevrier | Human Brain Mapping |
| 2007 | Chikazoe | Journal of Cognitive Neuroscience |
| 2007 | Choo | Alzheimer Disease and Associated Disorders |
| 2007 | Christoffels | Human Brain Mapping |
| 2007 | Creem-Regehr | NeuroImage |
| 2007 | Cross | Journal of Cognitive Neuroscience |
| 2007 | Curtis | Bipolar Disorders |
| 2007 | Deeley | Biological Psychiatry |
| 2007 | Devue | Brain Research |
| 2007 | Dichter | NeuroImage |
| 2007 | Dickstein | Bipolar Disorders |
| 2007 | Eddington | Journal of Cognitive Neuroscience |
| 2007 | Eippert | Human Brain Mapping |
| 2007 | Fu | American Journal of Psychiatry |
| 2007 | Galvan | Developmental Science |
| 2007 | Gamer | Human Brain Mapping |
| 2007 | Georgiou-Karistianis | Neuropsychologia |
| 2007 | Geuze | Archives of General Psychiatry |
| 2007 | Gizewski | NeuroImage |
| 2007 | Gobbini | Journal of Cognitive Neuroscience |
| 2007 | Goldstein | American Journal of Psychiatry |
| 2007 | Goossens | Psychiatry Research |
| 2007 | Gowen | NeuroImage |
| 2007 | Gu | NeuroImage |
| 2007 | Gur | Human Brain Mapping |
| 2007 | Haase | Journal of Neuroscience Methods |
| 2007 | Haller | European Journal of Neuroscience |
| 2007 | Harrington | Human Brain Mapping |
| 2007 | Hassabis | Journal of Neuroscience |
| 2007 | Herwig | NeuroImage |
| 2007 | Herwig | Psychiatry Research |
| 2007 | Hofer | Brain and Cognition |
| 2007 | Hou | Brain Research |
| 2007 | Huang | Neurobiology of Aging |
| 2007 | Hummel | Behavioural Brain Research |
| 2007 | Ilg | NeuroImage |
| 2007 | Jabbi | NeuroImage |
| 2007 | Jardri | NeuroImage |
| 2007 | Johnson | Cerebral Cortex |
| 2007 | Johnson | Journal of Neurology, Neurosurgery, and Psychiatry |
| 2007 | Kensinger | Neuropsychologia |
| 2007 | Kleber | NeuroImage |
| 2007 | Knutson | Neuron |
| 2007 | Kobayashi | Neuropsychologia |
| 2007 | Koch | Neuropsychologia |
| 2007 | Kramer | NeuroImage |
| 2007 | Kroliczak | Journal of Neurophysiology |
| 2007 | Lagopoulos | Journal of Psychiatry and Neuroscience |
| 2007 | Lagopoulos | Neuroreport |
| 2007 | Lamm | Journal of Cognitive Neuroscience |
| 2007 | Langenecker | Biological Psychiatry |
| 2007 | Lanius | Psychiatry Research |
| 2007 | Lee | Brain |
| 2007 | Lerner | Neurology |
| 2007 | Lissek | NeuroImage |
| 2007 | Liu | Human Brain Mapping |
| 2007 | Liu | Journal of Neuroscience |
| 2007 | Maihofner | European Journal of Neuroscience |
| 2007 | Malhi | Bipolar Disorders |
| 2007 | Malhi | Journal of Affective Disorders |
| 2007 | Mallol | Brain Research |
| 2007 | Marchand | Psychiatry Research |
| 2007 | Marco-Pallares | Neuroreport |
| 2007 | Marsh | NeuroImage |
| 2007 | Martin | Experimental Brain Research |
| 2007 | Mayer | NeuroImage |
| 2007 | Meister | PLoS ONE |
| 2007 | Milea | Neuroreport |
| 2007 | Mobbs | Science |
| 2007 | Moriguchi | Cerebral Cortex |
| 2007 | Murray | Journal of Neuroscience |
| 2007 | Nelson | Bipolar Disorders |
| 2007 | Neuner | Brain Research |
| 2007 | Ortigue | Journal of Cognitive Neuroscience |
| 2007 | Ortigue | NeuroImage |
| 2007 | Otten | Cerebral Cortex |
| 2007 | Parris | Journal of Cognitive Neuroscience |
| 2007 | Pavuluri | Biological Psychiatry |
| 2007 | Petrella | Radiology |
| 2007 | Plassmann | Journal of Neuroscience |
| 2007 | Postle | Cerebral Cortex |
| 2007 | Prado | Journal of Cognitive Neuroscience |
| 2007 | Qin | NeuroImage |
| 2007 | Ramasubbu | Canadian Journal of Psychiatry |
| 2007 | Rauch | Psychiatry Research |
| 2007 | Rektorova | Movement Disorders |
| 2007 | Rilling | Biological Psychiatry |
| 2007 | Roth | Biological Psychiatry |
| 2007 | Rothemund | NeuroImage |
| 2007 | Rotshtein | Journal of Cognitive Neuroscience |
| 2007 | Saarela | Cerebral Cortex |
| 2007 | Safron | Behavioral Neuroscience |
| 2007 | Sailer | NeuroImage |
| 2007 | Samanez-Larkin | Nature Neuroscience |
| 2007 | Samuraki | European Journal of Nuclear Medicine and Molecular Imaging |
| 2007 | Schnell | Journal of Psychiatric Research |
| 2007 | Schonberg | Journal of Neuroscience |
| 2007 | Schoning | Neuropsychologia |
| 2007 | Sheridan | Journal of the American Academy of Child and Adolescent Psychiatry |
| 2007 | Shibata | Brain Research |
| 2007 | Simmonds | Neuropsychologia |
| 2007 | Soderlund | NeuroImage |
| 2007 | Stark | NeuroImage |
| 2007 | Stern | Brain Research |
| 2007 | Stoeter | NeuroImage |
| 2007 | Straube | NeuroImage |
| 2007 | Suh | Brain Research |
| 2007 | Szameitat | European Journal of Neuroscience |
| 2007 | Szameitat | NeuroImage |
| 2007 | Thompson | Journal of Cognitive Neuroscience |
| 2007 | Tobler | Journal of Neurophysiology |
| 2007 | Tunik | Journal of Neurophysiology |
| 2007 | Walsh | Biological Psychiatry |
| 2007 | Wang | Archives of General Psychiatry |
| 2007 | Wang | NeuroImage |
| 2007 | Weiss | Psychiatry Research |
| 2007 | Wessa | American Journal of Psychiatry |
| 2007 | Winston | Neuropsychologia |
| 2007 | Wittmann | Experimental Brain Research |
| 2007 | Woodard | Journal of Cognitive Neuroscience |
| 2007 | Wrase | NeuroImage |
| 2007 | Yoo | Nature Neuroscience |
| 2007 | Yu | NeuroImage |
| 2007 | Zaki | Social Neuroscience |
| 2007 | de | Neuropsychologia |
| 2008 | Abe | Cerebral Cortex |
| 2008 | Aleman | PLoS ONE |
| 2008 | Asllani | Journal of Cerebral Blood Flow and Metabolism |
| 2008 | Bjork | Addiction |
| 2008 | Bokde | Psychiatry Research |
| 2008 | Bragulat | Alcoholism: Clinical and Experimental Research |
| 2008 | Brown | Cerebral Cortex |
| 2008 | Brune | Neuropsychologia |
| 2008 | Caffarra | Open Neuroimaging Journal |
| 2008 | Capek | Neuropsychologia |
| 2008 | Chandrasekhar | NeuroImage |
| 2008 | Ciumas | NeuroImage |
| 2008 | Coderre | Brain and Language |
| 2008 | Cooper | NeuroImage |
| 2008 | Deckersbach | Bipolar Disorders |
| 2008 | Del | European Journal of Nuclear Medicine and Molecular Imaging |
| 2008 | Dillon | Psychophysiology |
| 2008 | Dohnel | Neuropsychologia |
| 2008 | Drapier | Biological Psychiatry |
| 2008 | Drzezga | NeuroImage |
| 2008 | Dunsmoor | NeuroImage |
| 2008 | Eyler | Psychiatry Research |
| 2008 | Falconer | Journal of Psychiatry and Neuroscience |
| 2008 | Fitzgerald | Human Brain Mapping |
| 2008 | Fu | Biological Psychiatry |
| 2008 | Galati | NeuroImage |
| 2008 | Ghosh | Journal of Speech, Language, and Hearing Research |
| 2008 | Goldin | Biological Psychiatry |
| 2008 | Gomot | Brain |
| 2008 | Gundersen | Open Neuroimaging Journal |
| 2008 | Guroglu | NeuroImage |
| 2008 | Hampton | Proceedings of the National Academy of Sciences |
| 2008 | Hanakawa | Cerebral Cortex |
| 2008 | Hare | Journal of Neuroscience |
| 2008 | Harenski | Social Cognitive and Affective Neuroscience |
| 2008 | Hassel | Bipolar Disorders |
| 2008 | Helmchen | Human Brain Mapping |
| 2008 | Henderson | NeuroImage |
| 2008 | Herpertz | Journal of Child Psychology and Psychiatry |
| 2008 | Hoeft | Journal of Psychiatric Research |
| 2008 | Hoffman | Psychopharmacology |
| 2008 | Hooker | Neuropsychologia |
| 2008 | Hu | American Journal of Neuroradiology |
| 2008 | Huh | Journal of Sexual Medicine |
| 2008 | Hutchison | NeuroImage |
| 2008 | Iaria | Human Brain Mapping |
| 2008 | Iseki | NeuroImage |
| 2008 | Jogia | British Journal of Psychiatry |
| 2008 | Kanda | European Journal of Nuclear Medicine and Molecular Imaging |
| 2008 | Knutson | Biological Psychiatry |
| 2008 | Knutson | Neuroreport |
| 2008 | Koeneke | Behavioral and Brain Functions |
| 2008 | Koppelstaetter | NeuroImage |
| 2008 | Koshino | Cerebral Cortex |
| 2008 | Lee | Cerebral Cortex |
| 2008 | Li | Neuroscience |
| 2008 | Lissek | PLoS ONE |
| 2008 | LoPresti | Journal of Neuroscience |
| 2008 | Lowell | NeuroImage |
| 2008 | McKay | NeuroImage |
| 2008 | McNab | Neuropsychologia |
| 2008 | Mehnert | NeuroImage |
| 2008 | Mitterschiffthaler | Psychological Medicine |
| 2008 | Mohr | European Journal of Neuroscience |
| 2008 | Mulder | Journal of the American Academy of Child and Adolescent Psychiatry |
| 2008 | Munzert | Experimental Brain Research |
| 2008 | Noriuchi | Biological Psychiatry |
| 2008 | Payer | Drug and Alcohol Dependence |
| 2008 | Plassmann | Proceedings of the National Academy of Sciences |
| 2008 | Rao | NeuroImage |
| 2008 | Schiller | Journal of Neuroscience |
| 2008 | Schwartz | Brain |
| 2008 | Seseke | NeuroImage |
| 2008 | Shamosh | Psychological Science |
| 2008 | Shane | NeuroImage |
| 2008 | Shimomura | Turkish Neruosurgery |
| 2008 | Simmons | Journal of Physiology - Paris |
| 2008 | Sommer | Acta Neurobiologiae Experimentalis |
| 2008 | Spence | NeuroImage |
| 2008 | Strathearn | Pediatrics |
| 2008 | Strohle | NeuroImage |
| 2008 | Suskauer | Journal of Cognitive Neuroscience |
| 2008 | Tanaka | Journal of Neuroscience |
| 2008 | Thaut | PLoS ONE |
| 2008 | Tourville | NeuroImage |
| 2008 | Vanderwal | NeuroImage |
| 2008 | Vannini | Neurobiology of Disease |
| 2008 | Vinogradov | Cerebral Cortex |
| 2008 | Vossel | Neuropsychopharmacology |
| 2008 | Walter | NeuroImage |
| 2008 | Wittfoth | Brain Research |
| 2008 | Wong | Journal of Speech, Language, and Hearing Research |
| 2008 | Zheng | Journal of Cognitive Neuroscience |
| 2009 | Ballard | NeuroImage |
| 2009 | Beck | Biological Psychiatry |
| 2009 | Bennett | Neurotoxicology and Teratology |
| 2009 | Bickel | Journal of Neuroscience |
| 2009 | Blasi | Psychiatry Research |
| 2009 | Broome | British Journal of Psychiatry |
| 2009 | Chae | Neuroscience Letters |
| 2009 | Chikazoe | Cerebral Cortex |
| 2009 | Chikazoe | Journal of Neuroscience |
| 2009 | Christopoulos | Journal of Neuroscience |
| 2009 | Clark | Neuron |
| 2009 | Cooper | Social Cognitive and Affective Neuroscience |
| 2009 | Davis | Magnetic Resonance Imaging |
| 2009 | De | Neuropsychopharmacology |
| 2009 | Dichter | Journal of Affective Disorders |
| 2009 | Engelmann | PLoS ONE |
| 2009 | Ewbank | NeuroImage |
| 2009 | Glascher | Cerebral Cortex |
| 2009 | Goldstein | Journal of Neuroscience |
| 2009 | Guillot | Human Brain Mapping |
| 2009 | Hare | Science |
| 2009 | Hauk | European Journal of Neuroscience |
| 2009 | Hester | Neuropsychopharmacology |
| 2009 | Hooley | Psychiatry Research |
| 2009 | Hsu | Journal of Neuroscience |
| 2009 | Janata | Cerebral Cortex |
| 2009 | Kaladjian | Psychiatry Research |
| 2009 | Kato | Behavioral Neuroscience |
| 2009 | Keedy | Psychiatry Research |
| 2009 | Langbaum | NeuroImage |
| 2009 | Leaver | Journal of Neuroscience |
| 2009 | Lee | Brain and Cognition |
| 2009 | Li | Cerebral Cortex |
| 2009 | Liu | Journal of Magnetic Resonance Imaging |
| 2009 | Mak | Neuropsychologia |
| 2009 | Mandzia | Neurobiology of Aging |
| 2009 | Matthews | Psychiatry Research |
| 2009 | Mensebach | Psychiatry Research |
| 2009 | Mostofsky | Brain |
| 2009 | Obermann | NeuroImage |
| 2009 | Onur | Social Cognitive and Affective Neuroscience |
| 2009 | Remijnse | Psychological Medicine |
| 2009 | Robinson | Bipolar Disorders |
| 2009 | Salo | Biological Psychiatry |
| 2009 | Salvadore | Biological Psychiatry |
| 2009 | Schienle | Psychiatry Research |
| 2009 | Seifert | Journal of Neuroscience |
| 2009 | Shimada | Neurology |
| 2009 | Small | American Journal of Geriatric Psychiatry |
| 2009 | Smoski | Journal of Affective Disorders |
| 2009 | Spreckelmeyer | Social Cognitive and Affective Neuroscience |
| 2009 | Sripada | Neuroreport |
| 2009 | Summerfield | NeuroImage |
| 2009 | Tricomi | European Journal of Neuroscience |
| 2009 | Valentin | Journal of Neurophysiology |
| 2009 | Watson | Pain |
| 2009 | Welander-Vatn | Bipolar Disorders |
| 2009 | Werner | Journal of Affective Disorders |
| 2009 | Wilson | Brain |
| 2009 | Xu | Brain Research |
| 2009 | Xue | Cerebral Cortex |
| 2009 | Yakushev | NeuroImage |
| 2009 | Yang | Neuroreport |
| 2009 | Ye | NeuroImage |
| 2009 | Yoon | Neuroscience Letters |
| 2010 | Gruber | Human Brain Mapping |
| 2010 | Hare | Journal of Neuroscience |
| 2010 | Kumari | Schizophrenia Bulletin |
| 2010 | Mobascher | NeuroImage |
| 2010 | Righi | Journal of Cognitive Neuroscience |
| 2010 | Straube | Human Brain Mapping |

Table S2. Cluster 1

| **Cluster #** | **Volume (mm^3)** | **Weighted Center (x,y,z)** | | | **Extrema Value** | **x** | **y** | **z** | **Label** |  |  |  |  |  |  |  |  |
| --- | --- | --- | --- | --- | --- | --- | --- | --- | --- | --- | --- | --- | --- | --- | --- | --- | --- |
| **1** | 51088 | 29.34 | -4.57 | 16.79 | 0.35472572 | 54 | 14 | 8 | Right Cerebrum.Frontal Lobe.Precentral Gyrus.Gray Matter.Brodmann area 44 | | | | | | | |  |
|  |  |  |  |  | 0.3409929 | 52 | 2 | 22 | Right Cerebrum.Frontal Lobe.Inferior Frontal Gyrus.Gray Matter.Brodmann area 9 | | | | | | | |  |
|  |  |  |  |  | 0.31764123 | 34 | -36 | 40 | Right Cerebrum.Parietal Lobe.Inferior Parietal Lobule.Gray Matter.Brodmann area 40 | | | | | | | | |
|  |  |  |  |  | 0.28815386 | 50 | -30 | 42 | Right Cerebrum.Parietal Lobe.Inferior Parietal Lobule.Gray Matter.Brodmann area 40 | | | | | | | | |
|  |  |  |  |  | 0.28468683 | 8 | -18 | 8 | Right Cerebrum.Sub-lobar.Thalamus.Gray Matter.Medial Dorsal Nucleus | | | | | | |  |  |
|  |  |  |  |  | 0.27735242 | 60 | -4 | 0 | Right Cerebrum.Temporal Lobe.Superior Temporal Gyrus.Gray Matter.Brodmann area 22 | | | | | | | | |
|  |  |  |  |  | 0.2740218 | -14 | -18 | 8 | Left Cerebrum.Sub-lobar.Thalamus.Gray Matter.Ventral Posterior Medial Nucleus | | | | | | | |  |
|  |  |  |  |  | 0.27319053 | 20 | 34 | 38 | Right Cerebrum.Frontal Lobe.Middle Frontal Gyrus.Gray Matter.Brodmann area 8 | | | | | | | |  |
|  |  |  |  |  | 0.24700937 | 34 | 10 | 8 | Right Cerebrum.Sub-lobar.Insula.Gray Matter.Brodmann area 13 | | | | | | |  |  |
|  |  |  |  |  | 0.24270765 | 32 | 16 | 10 | Right Cerebrum.Sub-lobar.Insula.Gray Matter.Brodmann area 13 | | | | | | |  |  |
|  |  |  |  |  | 0.2261601 | 28 | 38 | 24 | Right Cerebrum.Frontal Lobe.Middle Frontal Gyrus.Gray Matter.Brodmann area 9 | | | | | | | |  |
|  |  |  |  |  | 0.21200919 | 8 | 6 | 6 | Right Cerebrum.Sub-lobar.Caudate.Gray Matter.Caudate Body | | | | | | |  |  |
|  |  |  |  |  | 0.21192431 | -24 | -10 | 8 | Left Cerebrum.Sub-lobar.Lentiform Nucleus.Gray Matter.Putamen | | | | | | |  |  |
|  |  |  |  |  | 0.19136506 | 54 | -36 | 28 | Right Cerebrum.Parietal Lobe.Inferior Parietal Lobule.Gray Matter.Brodmann area 40 | | | | | | | | |
|  |  |  |  |  | 0.18168029 | 52 | -14 | 18 | Right Cerebrum.Parietal Lobe.Postcentral Gyrus.Gray Matter.Brodmann area 43 | | | | | | | |  |
|  |  |  |  |  | 0.17129055 | -22 | -18 | -4 | Left Cerebrum.Sub-lobar.Lentiform Nucleus.Gray Matter.Lateral Globus Pallidus | | | | | | | |  |
|  |  |  |  |  | 0.1709219 | -18 | 0 | 14 | Left Cerebrum.Sub-lobar.Lentiform Nucleus.Gray Matter.Putamen | | | | | | |  |  |
|  |  |  |  |  | 0.16919923 | 26 | 0 | 8 | Right Cerebrum.Sub-lobar.Lentiform Nucleus.Gray Matter.Putamen | | | | | | |  |  |
|  |  |  |  |  | 0.16537574 | 26 | -54 | 42 | Right Cerebrum.Parietal Lobe.Superior Parietal Lobule.Gray Matter.Brodmann area 7 | | | | | | | | |
|  |  |  |  |  | 0.16331807 | 42 | 26 | 18 | Right Cerebrum.Frontal Lobe.Middle Frontal Gyrus.Gray Matter.Brodmann area 46 | | | | | | | |  |
|  |  |  |  |  | 0.15942419 | 36 | -10 | -8 | Right Cerebrum.Sub-lobar.Claustrum.Gray Matter.* | | | | | |  |  |  |
|  |  |  |  |  | 0.1432581 | 48 | -30 | 20 | Right Cerebrum.Sub-lobar.Insula.Gray Matter.Brodmann area 13 | | | | | | |  |  |
|  |  |  |  |  | 0.14028375 | 38 | 34 | 32 | Right Cerebrum.Frontal Lobe.Superior Frontal Gyrus.Gray Matter.Brodmann area 9 | | | | | | | |  |
|  |  |  |  |  | 0.13968942 | 54 | -38 | 18 | Right Cerebrum.Sub-lobar.Insula.Gray Matter.Brodmann area 13 | | | | | | |  |  |
|  |  |  |  |  | 0.13831975 | -6 | 4 | -6 | Left Cerebrum.Sub-lobar.*.Gray Matter.* | | | | |  |  |  |  |
|  |  |  |  |  | 0.12933046 | -10 | 12 | 2 | Left Cerebrum.Sub-lobar.Caudate.Gray Matter.Caudate Head | | | | | |  |  |  |
|  |  |  |  |  | 0.12204822 | 44 | 8 | 40 | Right Cerebrum.Frontal Lobe.Middle Frontal Gyrus.Gray Matter.Brodmann area 8 | | | | | | | |  |
|  |  |  |  |  | 0.115757525 | 48 | -46 | 34 | Right Cerebrum.Parietal Lobe.Supramarginal Gyrus.Gray Matter.Brodmann area 40 | | | | | | | |  |
|  |  |  |  |  | 0.1131533 | 34 | 16 | -8 | Right Cerebrum.Frontal Lobe.Extra-Nuclear.Gray Matter.Brodmann area 47 | | | | | | | |  |
| **2** | 41264 | -0.69 | -0.03 | 37.18 | 0.57670903 | 0 | -10 | 42 | Left Cerebrum.Limbic Lobe.Cingulate Gyrus.Gray Matter.Brodmann area 24 | | | | | | | |  |
|  |  |  |  |  | 0.42577025 | 0 | 2 | 44 | Left Cerebrum.Limbic Lobe.Cingulate Gyrus.Gray Matter.Brodmann area 24 | | | | | | | |  |
|  |  |  |  |  | 0.34827885 | -2 | 34 | 6 | Left Cerebrum.Limbic Lobe.Anterior Cingulate.Gray Matter.Brodmann area 24 | | | | | | | |  |
|  |  |  |  |  | 0.26127246 | -2 | -28 | 32 | Left Cerebrum.Limbic Lobe.Cingulate Gyrus.Gray Matter.Brodmann area 23 | | | | | | | |  |
|  |  |  |  |  | 0.22933494 | 6 | -32 | 42 | Right Cerebrum.Limbic Lobe.Cingulate Gyrus.Gray Matter.Brodmann area 31 | | | | | | | |  |
|  |  |  |  |  | 0.21232192 | -10 | 18 | 32 | Left Cerebrum.Limbic Lobe.Cingulate Gyrus.Gray Matter.Brodmann area 32 | | | | | | | |  |
|  |  |  |  |  | 0.20917095 | -2 | -46 | 32 | Left Cerebrum.Parietal Lobe.Precuneus.Gray Matter.Brodmann area 31 | | | | | | |  |  |
|  |  |  |  |  | 0.18975908 | 6 | -40 | 44 | Right Cerebrum.Parietal Lobe.Precuneus.Gray Matter.Brodmann area 7 | | | | | | |  |  |
|  |  |  |  |  | 0.18625067 | 4 | 20 | 34 | Right Cerebrum.Frontal Lobe.Cingulate Gyrus.Gray Matter.Brodmann area 32 | | | | | | | |  |
|  |  |  |  |  | 0.16204737 | -8 | 26 | 20 | Left Cerebrum.Limbic Lobe.Anterior Cingulate.Gray Matter.Brodmann area 32 | | | | | | | |  |
|  |  |  |  |  | 0.11074625 | 12 | 0 | 66 | Right Cerebrum.Frontal Lobe.Superior Frontal Gyrus.Gray Matter.Brodmann area 6 | | | | | | | |  |
|  |  |  |  |  | 0.10356236 | -4 | 30 | 30 | Left Cerebrum.Frontal Lobe.Medial Frontal Gyrus.Gray Matter.Brodmann area 9 | | | | | | | |  |
| **3** | 18088 | -45.85 | -38.58 | 23.8 | 0.3208526 | -50 | -36 | 12 | Left Cerebrum.Temporal Lobe.Superior Temporal Gyrus.Gray Matter.Brodmann area 41 | | | | | | | | |
|  |  |  |  |  | 0.2940158 | -50 | -40 | 28 | Left Cerebrum.Parietal Lobe.Inferior Parietal Lobule.Gray Matter.Brodmann area 40 | | | | | | | | |
|  |  |  |  |  | 0.28118598 | -40 | -60 | 12 | Left Cerebrum.Occipital Lobe.Middle Temporal Gyrus.Gray Matter.Brodmann area 19 | | | | | | | | |
|  |  |  |  |  | 0.27548102 | -44 | -26 | 30 | Left Cerebrum.Parietal Lobe.Postcentral Gyrus.Gray Matter.Brodmann area 2 | | | | | | | |  |
|  |  |  |  |  | 0.2190372 | -32 | -28 | 54 | Left Cerebrum.Frontal Lobe.Precentral Gyrus.Gray Matter.Brodmann area 4 | | | | | | | |  |
|  |  |  |  |  | 0.20213147 | -48 | -62 | 2 | Left Cerebrum.Temporal Lobe.Middle Temporal Gyrus.Gray Matter.* | | | | | | |  |  |
|  |  |  |  |  | 0.17133352 | -28 | -12 | 62 | Left Cerebrum.Frontal Lobe.Precentral Gyrus.Gray Matter.Brodmann area 6 | | | | | | | |  |
|  |  |  |  |  | 0.16238204 | -58 | -46 | 2 | Left Cerebrum.Temporal Lobe.Middle Temporal Gyrus.Gray Matter.Brodmann area 22 | | | | | | | | |
|  |  |  |  |  | 0.16072759 | -64 | -48 | 0 | Left Cerebrum.Temporal Lobe.Middle Temporal Gyrus.Gray Matter.Brodmann area 21 | | | | | | | | |
|  |  |  |  |  | 0.1465593 | -54 | -22 | 20 | Left Cerebrum.Parietal Lobe.Postcentral Gyrus.Gray Matter.Brodmann area 40 | | | | | | | |  |
|  |  |  |  |  | 0.14512444 | -40 | -48 | 28 | Left Cerebrum.Temporal Lobe.Superior Temporal Gyrus.Gray Matter.Brodmann area 39 | | | | | | | | |
| **4** | 16176 | -38.43 | 22.73 | 10.51 | 0.3197585 | -42 | 14 | 24 | Left Cerebrum.Frontal Lobe.Middle Frontal Gyrus.Gray Matter.Brodmann area 9 | | | | | | | |  |
|  |  |  |  |  | 0.3165678 | -34 | 40 | 20 | Left Cerebrum.Frontal Lobe.Middle Frontal Gyrus.Gray Matter.Brodmann area 10 | | | | | | | |  |
|  |  |  |  |  | 0.28572175 | -50 | 36 | 16 | Left Cerebrum.Frontal Lobe.Middle Frontal Gyrus.Gray Matter.* | | | | | | |  |  |
|  |  |  |  |  | 0.28373373 | -44 | 38 | -8 | Left Cerebrum.Frontal Lobe.Middle Frontal Gyrus.Gray Matter.Brodmann area 47 | | | | | | | |  |
|  |  |  |  |  | 0.23917173 | -34 | 20 | 10 | Left Cerebrum.Sub-lobar.Insula.Gray Matter.Brodmann area 13 | | | | | | |  |  |
|  |  |  |  |  | 0.23300779 | -42 | 6 | 12 | Left Cerebrum.Sub-lobar.Insula.Gray Matter.Brodmann area 13 | | | | | | |  |  |
|  |  |  |  |  | 0.22650279 | -34 | 36 | 10 | Left Cerebrum.Frontal Lobe.Middle Frontal Gyrus.Gray Matter.Brodmann area 10 | | | | | | | |  |
|  |  |  |  |  | 0.20069686 | -46 | 36 | 6 | Left Cerebrum.Frontal Lobe.Inferior Frontal Gyrus.Gray Matter.Brodmann area 46 | | | | | | | |  |
|  |  |  |  |  | 0.18265398 | -42 | 10 | -2 | Left Cerebrum.Sub-lobar.Insula.Gray Matter.Brodmann area 13 | | | | | | |  |  |
| **5** | 3792 | -47.1 | -6.4 | 31.91 | 0.18823415 | -40 | -6 | 36 | Left Cerebrum.Frontal Lobe.Precentral Gyrus.Gray Matter.Brodmann area 6 | | | | | | | |  |
|  |  |  |  |  | 0.15218931 | -58 | -8 | 24 | Left Cerebrum.Frontal Lobe.Precentral Gyrus.Gray Matter.Brodmann area 4 | | | | | | | |  |
|  |  |  |  |  | 0.13337594 | -40 | 0 | 48 | Left Cerebrum.Frontal Lobe.Middle Frontal Gyrus.Gray Matter.Brodmann area 6 | | | | | | | |  |
| **6** | 2968 | 15.22 | -49.64 | -21.2 | 0.23089045 | 14 | -48 | -20 | Right Cerebellum.Anterior Lobe.*.Gray Matter.Dentate | | | | | |  |  |  |
|  |  |  |  |  | 0.1771203 | 24 | -46 | -26 | Right Cerebellum.Anterior Lobe.*.Gray Matter.* | | | | |  |  |  |  |
| **7** | 2136 | 35.22 | -82.65 | -12.6 | 0.2763556 | 36 | -90 | -12 | Right Cerebrum.Occipital Lobe.Inferior Occipital Gyrus.Gray Matter.Brodmann area 18 | | | | | | | | |
|  |  |  |  |  | 0.16006003 | 36 | -76 | -14 | Right Cerebellum.Posterior Lobe.Declive.Gray Matter.* | | | | | |  |  |  |
|  |  |  |  |  | 0.10934589 | 30 | -82 | -6 | Right Cerebrum.Occipital Lobe.Middle Occipital Gyrus.Gray Matter.Brodmann area 18 | | | | | | | | |
| **8** | 1792 | 9.13 | -19.89 | -17.85 | 0.2826325 | 12 | -20 | -16 | No Gray Matter found | | |  |  |  |  |  |  |
|  |  |  |  |  | 0.16510497 | 4 | -20 | -22 | No Gray Matter found | | |  |  |  |  |  |  |
| **9** | 1680 | -45.66 | -45.28 | -9.89 | 0.28253806 | -48 | -46 | -12 | Left Cerebrum.Temporal Lobe.Fusiform Gyrus.Gray Matter.Brodmann area 37 | | | | | | | |  |
| **10** | 1520 | -28.57 | -51.36 | -31.23 | 0.1702872 | -28 | -54 | -30 | Left Cerebellum.Anterior Lobe.*.Gray Matter.* | | | | |  |  |  |  |
|  |  |  |  |  | 0.14008215 | -28 | -46 | -40 | Left Cerebellum.Posterior Lobe.Cerebellar Tonsil.Gray Matter.* | | | | | | |  |  |
|  |  |  |  |  | 0.102930374 | -36 | -46 | -26 | Left Cerebellum.Anterior Lobe.Culmen.Gray Matter.* | | | | | |  |  |  |
| **11** | 1472 | -52.51 | -10.74 | -12.93 | 0.3219365 | -54 | -10 | -12 | Left Cerebrum.Temporal Lobe.Middle Temporal Gyrus.Gray Matter.Brodmann area 21 | | | | | | | | |
| **12** | 1384 | -26.29 | -89.51 | -0.45 | 0.31313747 | -26 | -90 | 0 | Left Cerebrum.Occipital Lobe.Inferior Occipital Gyrus.Gray Matter.Brodmann area 18 | | | | | | | | |
| **13** | 1328 | 33.69 | -15.74 | -28.84 | 0.2879045 | 36 | -16 | -28 | Right Cerebrum.Limbic Lobe.Uncus.Gray Matter.Brodmann area 20 | | | | | | |  |  |
| **14** | 1312 | -37.77 | -13.89 | -24.21 | 0.27688435 | -40 | -16 | -24 | Left Cerebrum.Temporal Lobe.Fusiform Gyrus.Gray Matter.Brodmann area 20 | | | | | | | |  |
|  |  |  |  |  | 0.1345236 | -32 | -6 | -26 | Left Cerebrum.Limbic Lobe.Uncus.Gray Matter.Brodmann area 28 | | | | | | |  |  |
| **15** | 1248 | 42.38 | -65.64 | 0.12 | 0.29409388 | 42 | -66 | 0 | Right Cerebrum.Occipital Lobe.Inferior Temporal Gyrus.Gray Matter.* | | | | | | |  |  |
| **16** | 1208 | 24.31 | -10.07 | 51.97 | 0.1903942 | 24 | -12 | 52 | Right Cerebrum.Frontal Lobe.Middle Frontal Gyrus.Gray Matter.Brodmann area 6 | | | | | | | |  |
| **17** | 1192 | -25.02 | -61.55 | 45.55 | 0.1974504 | -26 | -60 | 46 | Left Cerebrum.Parietal Lobe.Superior Parietal Lobule.Gray Matter.Brodmann area 7 | | | | | | | | |
| **18** | 1088 | -20.85 | 36.82 | 39.84 | 0.21861514 | -24 | 34 | 38 | Left Cerebrum.Frontal Lobe.Middle Frontal Gyrus.Gray Matter.Brodmann area 8 | | | | | | | |  |
|  |  |  |  |  | 0.14087988 | -16 | 42 | 46 | Left Cerebrum.Frontal Lobe.Superior Frontal Gyrus.Gray Matter.Brodmann area 8 | | | | | | | |  |
| **19** | 848 | 17.76 | -57.98 | 4.09 | 0.27595678 | 18 | -58 | 4 | Right Cerebrum.Limbic Lobe.Posterior Cingulate.Gray Matter.Brodmann area 30 | | | | | | | |  |
| **20** | 808 | -29.86 | 9.99 | -27.77 | 0.27442774 | -30 | 10 | -28 | Left Cerebrum.Temporal Lobe.Superior Temporal Gyrus.Gray Matter.Brodmann area 38 | | | | | | | | |
| **21** | 760 | 52 | 6.07 | -19.92 | 0.2723092 | 52 | 6 | -20 | Right Cerebrum.Temporal Lobe.Middle Temporal Gyrus.Gray Matter.Brodmann area 21 | | | | | | | | |
| **22** | 624 | -11.31 | -92.55 | -7.63 | 0.16140518 | -12 | -92 | -8 | Left Cerebrum.Occipital Lobe.Inferior Occipital Gyrus.Gray Matter.Brodmann area 17 | | | | | | | | |
| **23** | 616 | -6.97 | -60.65 | 10.58 | 0.16400684 | -8 | -60 | 10 | Left Cerebrum.Occipital Lobe.Cuneus.Gray Matter.Brodmann area 30 | | | | | | |  |  |
| **24** | 512 | 21.92 | -80.44 | -15.96 | 0.15317407 | 22 | -80 | -16 | Right Cerebellum.Posterior Lobe.Declive.Gray Matter.* | | | | | |  |  |  |
| **25** | 496 | 50.47 | -51.52 | 5.86 | 0.15724798 | 50 | -52 | 6 | Right Cerebrum.Temporal Lobe.Superior Temporal Gyrus.Gray Matter.Brodmann area 39 | | | | | | | | |
| **26** | 488 | -4.93 | -34.27 | -10.28 | 0.18673804 | -4 | -34 | -10 | Left Cerebellum.Anterior Lobe.Culmen.Gray Matter.* | | | | | |  |  |  |
| **27** | 408 | 39.46 | 38.13 | -6.3 | 0.15419625 | 40 | 38 | -6 | Right Cerebrum.Frontal Lobe.Middle Frontal Gyrus.Gray Matter.Brodmann area 47 | | | | | | | |  |
| **28** | 376 | -18.18 | -54.41 | -12.65 | 0.1475745 | -18 | -54 | -12 | Left Cerebellum.Posterior Lobe.Declive.Gray Matter.* | | | | | |  |  |  |
| **29** | 288 | -62.62 | -27.53 | 27.49 | 0.1425739 | -64 | -28 | 28 | Left Cerebrum.Parietal Lobe.Inferior Parietal Lobule.Gray Matter.Brodmann area 40 | | | | | | | | |
| **30** | 280 | -48 | -30.05 | -8.87 | 0.13860872 | -50 | -30 | -8 | Left Cerebrum.Temporal Lobe.Middle Temporal Gyrus.Gray Matter.Brodmann area 21 | | | | | | | | |
| **31** | 280 | -3.49 | -71.99 | 31.12 | 0.12992951 | -4 | -72 | 30 | Left Cerebrum.Occipital Lobe.Cuneus.Gray Matter.Brodmann area 7 | | | | | | |  |  |
| **32** | 272 | 21.21 | 3.13 | 39.1 | 0.13593493 | 22 | 4 | 38 | Right Cerebrum.Limbic Lobe.Cingulate Gyrus.Gray Matter.Brodmann area 24 | | | | | | | |  |
| **33** | 272 | -31.86 | -46.3 | 41.22 | 0.122957885 | -32 | -46 | 42 | Left Cerebrum.Parietal Lobe.Inferior Parietal Lobule.Gray Matter.Brodmann area 40 | | | | | | | | |
| **34** | 264 | -17.94 | -42.07 | 22.82 | 0.13488427 | -18 | -42 | 22 | Left Cerebrum.Limbic Lobe.Cingulate Gyrus.Gray Matter.Brodmann area 31 | | | | | | | |  |
| **35** | 256 | -43.91 | -20.01 | 48.09 | 0.13245974 | -44 | -20 | 48 | Left Cerebrum.Parietal Lobe.Postcentral Gyrus.Gray Matter.Brodmann area 3 | | | | | | | |  |
| **36** | 240 | 27.99 | -30.25 | 62.17 | 0.14231989 | 28 | -30 | 62 | Right Cerebrum.Parietal Lobe.Postcentral Gyrus.Gray Matter.Brodmann area 3 | | | | | | | |  |
| **37** | 208 | -41.54 | -69.07 | 26.56 | 0.123240426 | -42 | -68 | 26 | Left Cerebrum.Temporal Lobe.Middle Temporal Gyrus.Gray Matter.Brodmann area 39 | | | | | | | | |
| **38** | 184 | -62.43 | -15.64 | 3.69 | 0.14076985 | -64 | -16 | 4 | Left Cerebrum.Temporal Lobe.Superior Temporal Gyrus.Gray Matter.Brodmann area 22 | | | | | | | | |
| **39** | 112 | -10.13 | -46.28 | -21.58 | 0.12232023 | -10 | -46 | -22 | Left Cerebellum.Anterior Lobe.Culmen.Gray Matter.* | | | | | |  |  |  |
| **40** | 96 | 39 | 16.33 | 31.65 | 0.11482681 | 40 | 16 | 32 | Right Cerebrum.Frontal Lobe.Middle Frontal Gyrus.Gray Matter.Brodmann area 9 | | | | | | | |  |
| **41** | 96 | 15 | -71 | 44.03 | 0.11758261 | 16 | -72 | 44 | Right Cerebrum.Parietal Lobe.Precuneus.Gray Matter.Brodmann area 7 | | | | | | |  |  |
| **42** | 88 | 12.55 | -16.37 | 67.09 | 0.11315 | 12 | -16 | 68 | Right Cerebrum.Frontal Lobe.Precentral Gyrus.Gray Matter.Brodmann area 6 | | | | | | | |  |
| **43** | 64 | 27 | -62.99 | -37 | 0.106256485 | 28 | -62 | -38 | Right Cerebellum.Posterior Lobe.Cerebellar Tonsil.Gray Matter.* | | | | | | |  |  |
| **44** | 64 | -43 | -21.01 | -7 | 0.10774302 | -44 | -22 | -8 | Left Cerebrum.Temporal Lobe.Superior Temporal Gyrus.Gray Matter.Brodmann area 22 | | | | | | | | |
| **45** | 48 | 38.33 | -39.67 | 28.66 | 0.11099143 | 38 | -40 | 28 | No Gray Matter found | | |  |  |  |  |  |  |
| **46** | 32 | -33.05 | 55 | -7 | 0.10985723 | -34 | 54 | -8 | Left Cerebrum.Frontal Lobe.Middle Frontal Gyrus.Gray Matter.Brodmann area 10 | | | | | | | |  |
| **47** | 32 | 14.99 | 20.99 | 10 | 0.10481722 | 14 | 20 | 10 | Right Cerebrum.Sub-lobar.Caudate.Gray Matter.Caudate Body | | | | | | |  |  |
| **48** | 32 | -40.01 | -20.99 | 18 | 0.103047885 | -40 | -20 | 18 | Left Cerebrum.Sub-lobar.Insula.Gray Matter.Brodmann area 13 | | | | | | |  |  |
| **49** | 32 | -18.49 | -74.5 | 23.52 | 0.111575134 | -18 | -74 | 24 | Left Cerebrum.Parietal Lobe.Precuneus.Gray Matter.Brodmann area 31 | | | | | | |  |  |

Table S3. Cluster 2

Table S4. Cluster 3

Figure S1


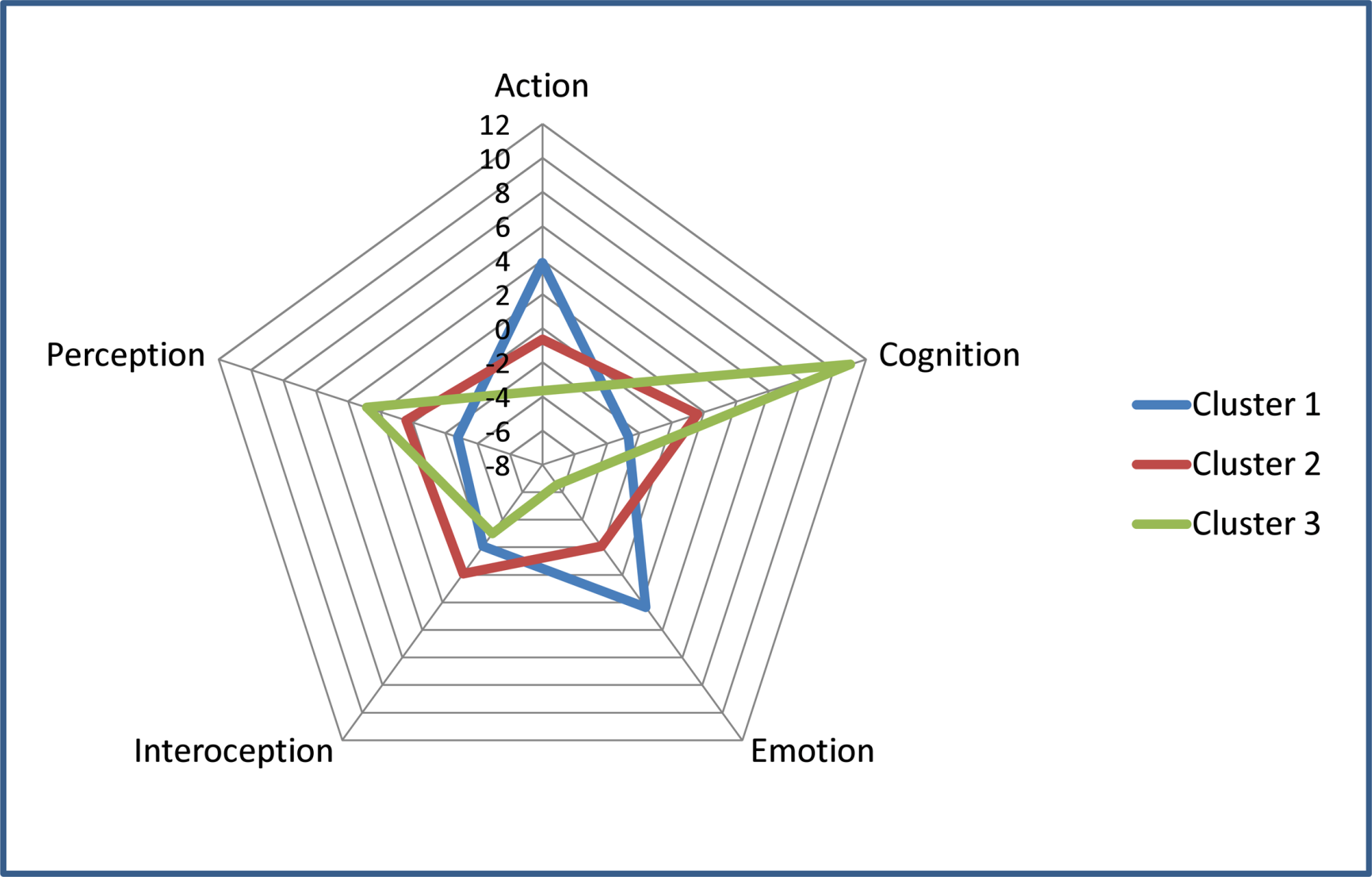


Behavioral classes that activate each cluster. The graph shows the number of papers (percent of difference from the mean) that statistically produced activations in each cluster. Only behavioral classes present in at least 5% of the papers were included (first term). All the behavioral classes (first term) that exceeded 5% of the mean are represented.

Figure S2


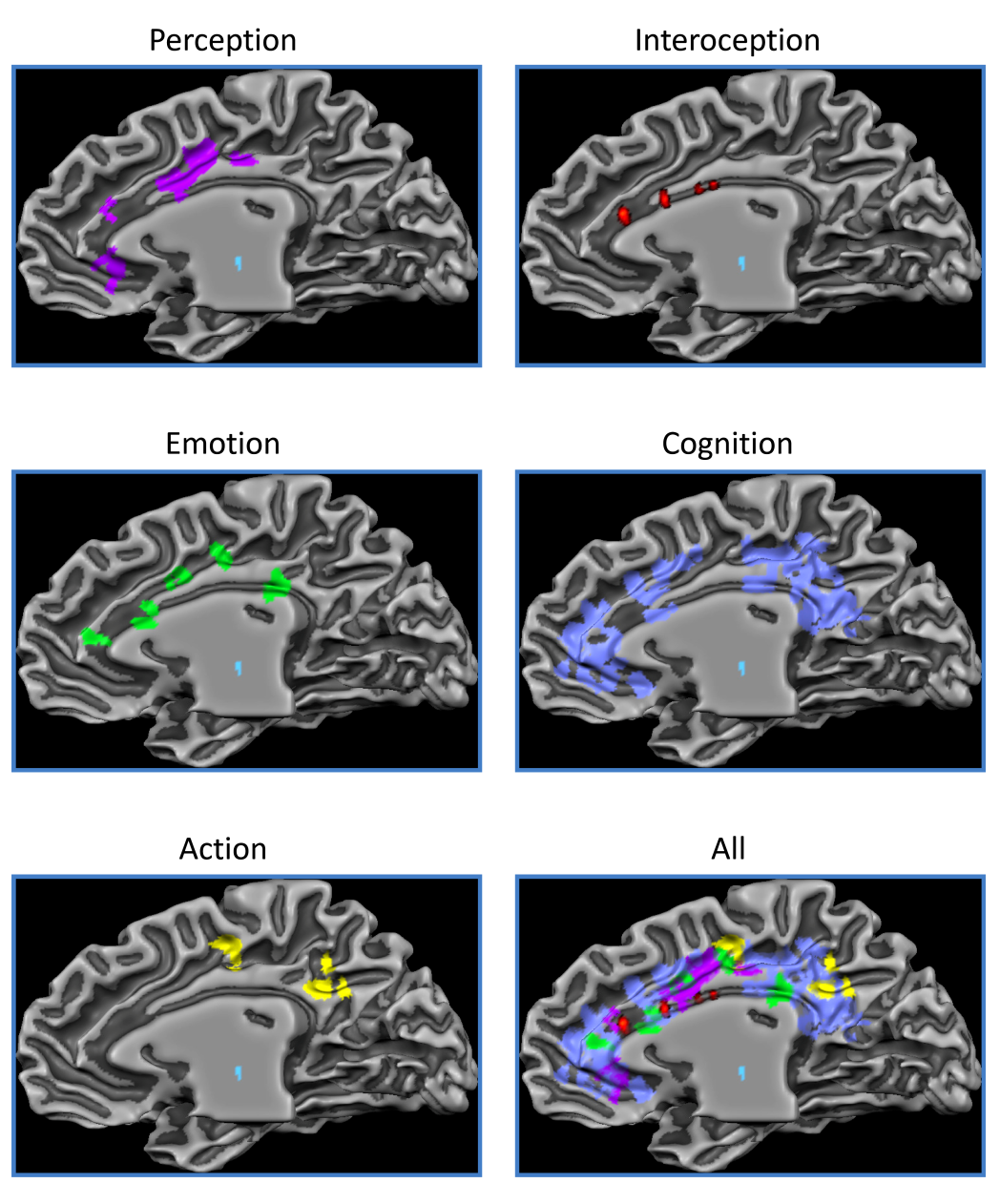


Winner-Take-All map of the behavioral-related density of foci. We performed a separate density analysis for each of the five prevalent behavioral classes in the cingulate cortex. The colors represent the areas where the density of one class is greater than the density of other classes

Figure S3


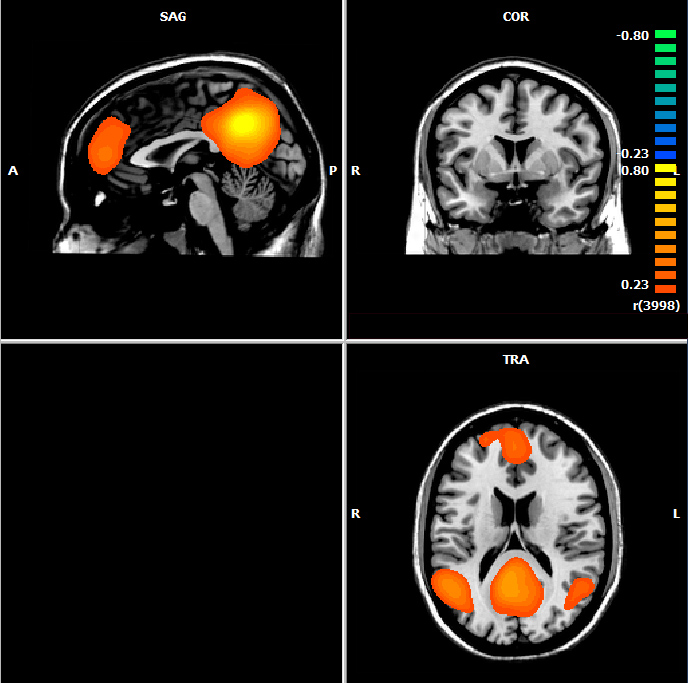


Activation for the resting state protocol

Figure S4


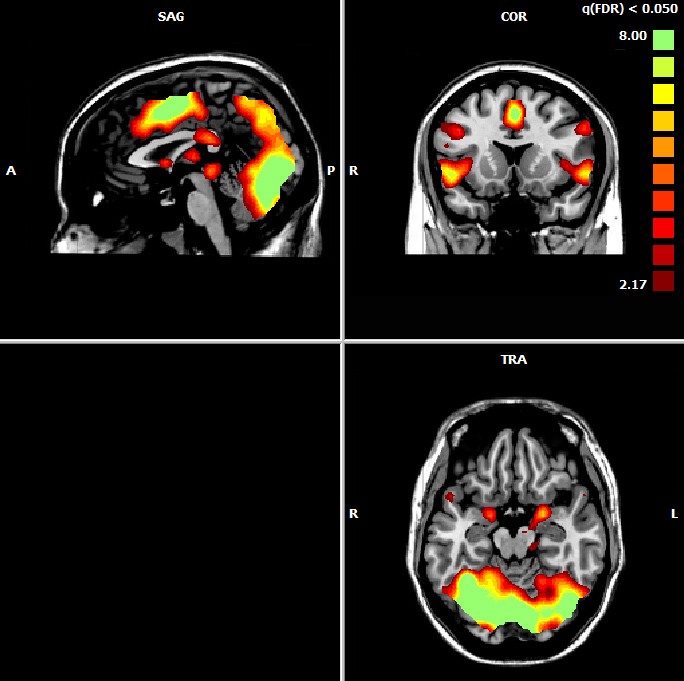


Activations for the emotion paradigm

Figure S5


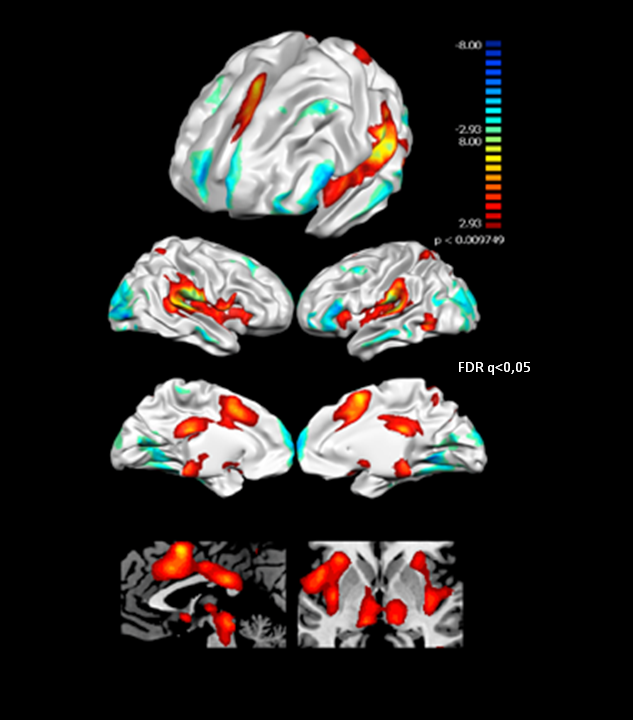


Activations for the pain paradigm

Figure S6


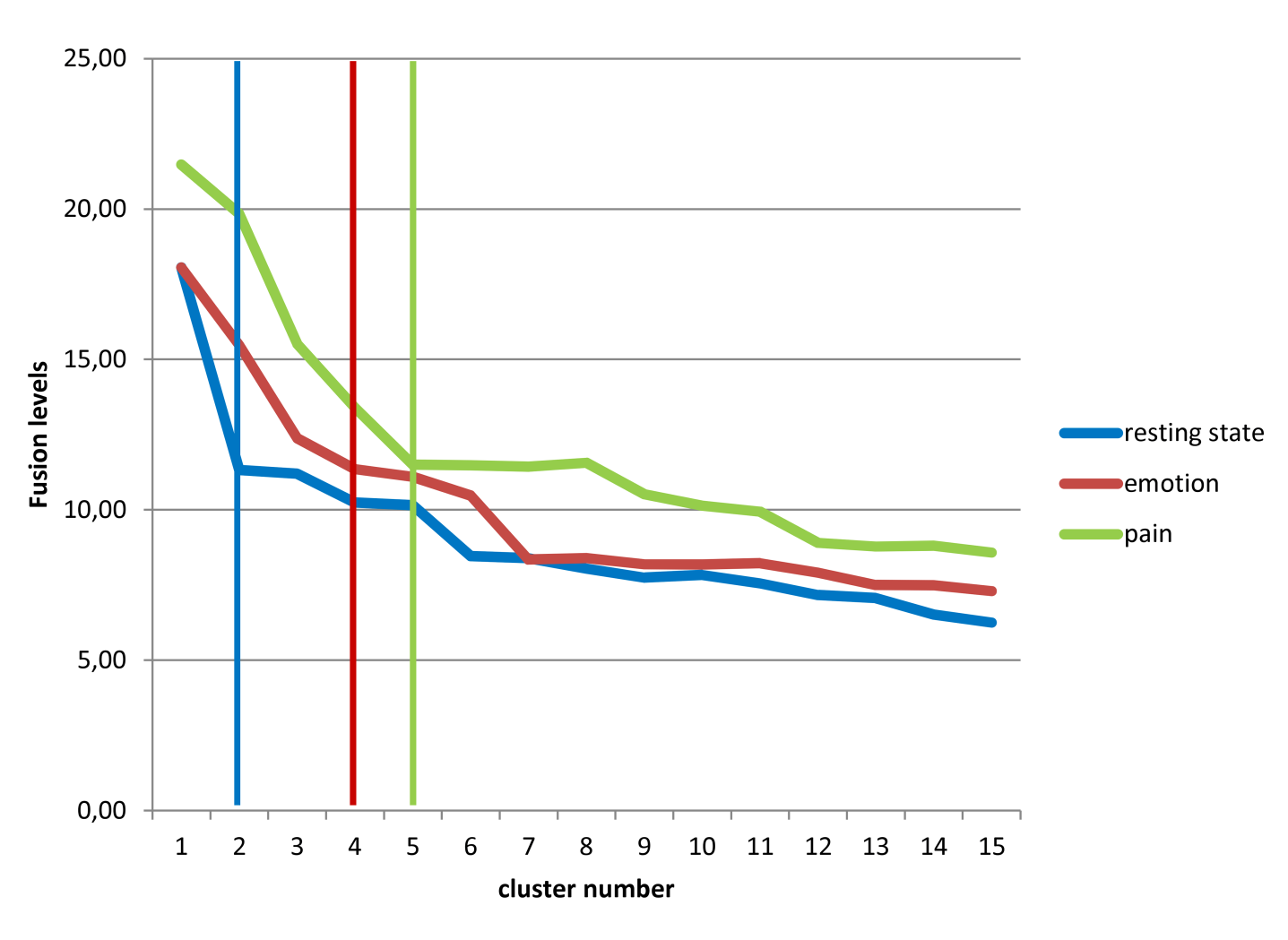


Validation of the number of clusters by the stopping criterion
